# Conserving the functional and phylogenetic trees of life of European tetrapods

**DOI:** 10.1098/rstb.2014.0005

**Published:** 2015-02-19

**Authors:** Wilfried Thuiller, Luigi Maiorano, Florent Mazel, François Guilhaumon, Gentile Francesco Ficetola, Sébastien Lavergne, Julien Renaud, Cristina Roquet, David Mouillot

**Affiliations:** 1 LECA, Université Grenoble Alpes, Grenoble 38000, France; 2 LECA, CNRS, Grenoble 38000, France; 3 Dipartimento di Biologia e Biotecnologie ‘Charles Darwin’, Università di Roma ‘La Sapienza’, Roma 00185, Italy; 4 Laboratoire ECOSYM, UMR 5119 CNRS-UM2-IRD-IFREMER, Place Eugène Bataillon cc 93, Montpellier 34095, France

**Keywords:** gap analysis, evolutionary distinctiveness, functional distinctiveness, protected areas, endemics

## Abstract

Protected areas (PAs) are pivotal tools for biodiversity conservation on the Earth. Europe has had an extensive protection system since Natura 2000 areas were created in parallel with traditional parks and reserves. However, the extent to which this system covers not only taxonomic diversity but also other biodiversity facets, such as evolutionary history and functional diversity, has never been evaluated. Using high-resolution distribution data of all European tetrapods together with dated molecular phylogenies and detailed trait information, we first tested whether the existing European protection system effectively covers all species and in particular, those with the highest evolutionary or functional distinctiveness. We then tested the ability of PAs to protect the entire tetrapod phylogenetic and functional trees of life by mapping species' target achievements along the internal branches of these two trees. We found that the current system is adequately representative in terms of the evolutionary history of amphibians while it fails for the rest. However, the most functionally distinct species were better represented than they would be under random conservation efforts. These results imply better protection of the tetrapod functional tree of life, which could help to ensure long-term functioning of the ecosystem, potentially at the expense of conserving evolutionary history.

## 1. Introduction

Protecting rare, threatened or emblematic species has always guided conservation strategies [1,2]. To this end, gap analyses have traditionally been used to identify priorities in reinforcing the effectiveness of protected area (PA) systems for sustaining viable populations and ensuring the local persistence of those populations while also favouring the protection of the remaining species [3,4]. Gap analysis is essentially an investigation of the overlap between the distributions of species and given PAs and is used to define the extent to which species are represented within PAs. This is then compared to prescribed species' targets, usually defined in order to ensure local persistence and based on species ranges [5].

As it has been recognized that the diversity of biological features held by different species deserves attention beyond the number and the status of species [6–8], conservationists have explored how effective PA networks are in protecting phylogenetic and functional diversity [9–11]. Through the course of evolution, species diversification and extinction lead to having species representing different amounts of evolutionary history, some species being unique in representing long evolutionary history (i.e. echidna for mammals [12]). The extinction of a species in an old, monotypic or species-poor clade would thus lead to a greater loss of phylogenetic diversity than that of a species belonging to a young lineage with many close relatives [13]. By combining a metric that measured mammal species evolutionary distinctiveness (i.e. contribution of a species to the overall tree of life) together with extinction risk, the EDGE framework was the first to assess the ability of PA networks to protect those distinct and endangered species [14]. However, this approach has been applied neither to other groups nor to large PA networks and has never been extended to other biological features.

Even though species evolutionary distinctiveness is key to prioritizing conservation efforts, it overlooks other important biological features such as functional traits that support ecosystem functioning and resilience to environmental changes [15,16]. In fact, species that support the most distinct combinations of traits, i.e. which have the highest functional distinctiveness, are not necessarily the most evolutionary distinct because species traits result from interplays between the history of natural selection, adaptive convergence and phylogenetic conservatism across time [17,18]. There is, therefore, an urgent need to assess (either empirically or theoretically) whether evolutionary distinct species are also functionally distinct in order to better prioritize conservation efforts to target the rarest of the rare [19]. A recent analysis has indeed highlighted that the most distinct combination of traits are predominantly supported by rare species [16]. If those rare species are not adequately protected, some particular functions will be highly vulnerable, potentially imperilling particular ecosystem processes [20]. The same applies when species with particular evolutionary history or functionality are also endemic of the area under investigation.

In terms of conservation efforts, Europe has one of the most extensive PA networks around the world. In addition to its traditional national parks and reserves, Europe also has Natura 2000 areas (in the countries involved in EU28 only), which were created to ensure the long-term persistence of species and habitats [21]. Natura 2000 is based on special protection areas, classified under the Birds Directive to protect important sites for rare and vulnerable birds, and special areas of conservation classified under the Habitats Directive to protect rare and vulnerable animals, plants and habitats [21].

A recent study has shown that although species representation within Natura 2000 is uneven, the network is relatively efficient in protecting target species (i.e. species with a specific conservation focus) and minimizes the number of gap species, e.g. species with no protected range [22]. However, this representation may be challenged by climate change [23] and whether the phylogenetic and functional diversity is adequately protected remains unknown.

In this paper, we have conducted a comprehensive gap analysis to assess the effectiveness of the European PA network's (national parks, reserves and Natura 2000) representativeness in terms of two overlooked facets of biodiversity in addition to the number of species: the breadth of evolutionary history and the functional diversity of most animal tetrapods occurring within Europe. The defined species-specific conservation targets are inversely proportional to species range sizes, so we first tested to see whether the most evolutionary and functionally distinct species are well-represented relative to other species. This analysis was carried out for all species occurring in Europe, with a specific focus on species endemic to Europe. By calculating how close species were to their conservation targets, we were then able to analyse how representative the European PA system is in terms of overall tetrapod phylogenetic and functional diversity. We compared the results to those obtained from a null model simulating random conservation efforts across species, independently of their biological features.

## 2. Material and methods

### (a) Study area and protected area networks

The study area included the entire European sub-continent plus Anatolia in order to include a complete picture of the North Mediterranean coast (hereafter: Europe; electronic supplementary material, figure S1). We conducted the analyses by combining two PA networks: PAs belonging to the International Union for Conservation of Nature (IUCN) category I and II from the World Database on Protected Areas (http://protectedplanet.net/) and all Natura 2000 areas (http://www.eea.europa.eu/) for the EU28 within the entire European sub-continent.

### (b) Species distributions

We collected data on 288 mammals, 509 birds, 104 amphibians and 239 squamate reptiles. These datasets were compiled from Maiorano *et al.* [24]. For mammals and amphibians, the main data sources were extent of occurrences (EOOs) compiled by the IUCN Global Mammal Assessment and Global Amphibian Assessment [25]. For bird species, the EOOs available from Hagemeijer & Blair [26] were combined with those available from the BWP*i*2.0.1 DVD-ROM (Birds of the Western Palearctic interactive 2006, v. 2.0.1). For squamates, the main data source for EOOs were Sindaco & Jeremcenko [27] and Sindaco *et al*. [28], integrated for a few species with the Global Reptile Assessment [25].

For the four groups, the EOOs were then refined using habitat preferences for all species, obtained from expert opinion and published literature [24]. The collected data were used to assign a suitability score (0, unsuitable, 1, suitable habitat) to each of the 46 GlobCover land-use/land-cover classes (300 m resolution). Scores were used to remove unsuitable cells (scored 0) and to refine EOOs of the four species groups (no presence data were added, but false presence data were removed [29].

The EOO for all species of all groups was thereby refined to 300 m resolution and was then evaluated against field data for 34 species of amphibians (37% of the 92 amphibians considered in the final species list; see paragraph below), 272 species of birds (71.4% of the 381 breeding birds considered in the final species list), 88 mammals (33.8% of the 246 mammals considered in the final species list) and 33 squamates (16.8% of the 196 squamates considered in the final species list). All refined EOOs evaluated for amphibians and mammals performed significantly better than random ones, while the percentage was lower for squamates (97.1% of the refined EOOs performing better than random) and breeding birds (96.3% of the refined EOOs performing better than random). Full details of the model evaluation procedure are provided in the electronic supplementary material and in Maiorano *et al.* [24].

For all species, we also calculated the proportion of their complete global range found within in Europe by dividing the surface area of the European portion of their distribution range (non-refined EOO) by the area of their global range. Data on global distributions were taken from IUCN Global Mammal Assessment and Global Amphibian Assessment [25], from [30], and from Sindaco & Jeremcenko [27] and Sindaco *et al.* [28]. We coined this metric as the endemicity status with a scale ranging theoretically from 0%, when the species does not occur in Europe, to 100%, when the species is strictly endemic to Europe.

### (c) Phylogenetic trees

Phylogenetic data for mammals were based on the updated supertree of Fritz & Purvis [31]. We used 100 fully resolved phylogenetic trees, where polytomies were randomly resolved using a birth–death model to simulate branch lengths [32]. We updated these phylogenetic trees by replacing the Carnivora clade in this phylogeny with a highly resolved supertree published more recently [33].

For birds and amphibians, we extracted the 100 dated and fully dichotomous phylogenetic trees from Roquet *et al.* [34] and Zupan *et al*. [10], respectively.

For squamates, phylogenetic inference was based on DNA sequence data from seven nuclear (BDNF, c-mos, NT3, PDC, R35, RAG-1 and RAG-2) and six mithocondrial loci (12S, 16S, COI, cytB, ND2 and ND4), which were extracted from GenBank with PHLAWD [35]. We included three levels of outgroup taxa: *Sphenodon punctata* (closest living relative to Squamata); European turtles, two crocodilians (*Alligator* and *Crocodylus*) and two birds (*Dromaius* and *Gallus*); and finally, two mammals (*Mus* and *Pan*). DNA sequences were aligned with MAFFT [36] and ambiguous regions were trimmed with trimAl [37]. A phylogenetic analysis was conducted with RaxML [38] to search for 100 maximum-likelihood trees, while applying a family tree constraint based on Pyron *et al.* [39]. The 100 trees were dated with penalized-likelihood as implemented in r8s [40]; we constrained five nodes based on fossil information extracted from Mulcahy *et al.* [41].

### (d) Functional traits and functional trees

We chose to restrict our analyses to comparable traits between the four groups. We thus selected traits that represent informative niche dimensions. These were body mass/body length, diet type, feeding behaviour, nesting position, reproduction and activity (see the electronic supplementary material, Functional trait database). These traits are known to relate to ecosystem functioning because they summarize or are linked to trophic interactions and resource acquisition [42–44] and were selected for this reason.

For birds, trait information was extracted from [18], this source mostly obtained its data from the Handbook of the Birds of the Western Palaearctic [45]. Missing species and data were gathered from species-specific publications and Internet websites dealing with avifauna. Traits for mammals, squamates and amphibians were extracted from various sources and compiled by the authors (see the electronic supplementary material).

To analyse the amount of functional diversity retained by species in the same way as the amount of phylogenetic diversity that had been analysed, we built up functional trees of life derived from functional trait distances between pairs of species. We log-transformed and normalized body mass/body length to a value between 0 and 1 prior to all analyses. We used a mixed-variable coefficient of distance that generalizes Gower's coefficient of distance and allows various types of variables to be treated when calculating distances [46]. Euclidean distance was used for body mass and body length, while the Sørensen distance [47] (S7 coefficient of Gower & Legendre [48], function *dist.ktab* in *ade4*) was used for all remaining binary traits. We then used hierarchical clustering to build a dendrogram of all species in functional-trait space, employing an average agglomeration method (UPGMA, function *hclust*) [49]. The use of functional dendrograms is somewhat controversial because it is difficult to fully grasp the ecological nature of hierarchy [50]. It is relatively logical, however, to consider feeding behaviour and diet to be nested because a carnivore that eats large prey generally hunts to catch it. We checked the extent to which functional variation was hierarchical by correlating the phenetic distances (pairwise distances across the dendrogram) with the pairwise distances in the initial distance matrix used to construct the dendrogram. Mantel tests using 9999 randomizations showed very high and significant correlations for the four groups (amphibians: 86%, birds: 82%, mammals: 80% and squamates: 87%) highlighting here that the use of a functional dendrogram did not lead to a strong distortion of the functional space [49].

Given that trait and/or phylogenetic information were not available for all species, we finally restricted our analyses to 381 birds, 246 mammals, 196 squamates and 92 amphibians. We retained species for examination where all traits were available or where a maximum of one trait was not available. Out of the 915 species analysed, 280 were strictly endemic to Europe.

### (e) Gap analysis

The major advantage of using EOOs refined at 300 m, to represent the amount of suitable habitat within the coarse resolution EOO, was that this provided an accurate match when compared with the PAs. We were thus able to lay the refined EOO for each species over the European PAs and thereby determine what proportion of their current European range was represented within the PA system.

One of the most subjective aspects of gap analyses is in the definition of species-specific representation targets. In terms of range within the PA network, this meant the level above which we would consider a species to be adequately covered. These specific targets are necessarily related to species range sizes as restricted species need more coverage than widespread ones to avoid extinction [51]. Accordingly, species-specific conservation targets, or the proportion of species geographical ranges that had to fall within the PA system in order to ensure their persistence, were set to be inversely proportional to log-transformed European species’ range sizes. Hence, species with restricted ranges required 100% of their range to be covered, whereas widespread species only required 10% [52]. We fitted a linear regression between these two extremes to define the target for the remaining species (see the electronic supplementary material, figure S2). We conducted the species-specific target estimations for the four groups separately. This had the advantage of enabling us to take into consideration the fact that the minimum range size for a reptile is different than for a bird species, for instance. This approach assumed that the species with the largest range is in an optimal situation and requires a minimal level of protection (approx. 10%).

We then extracted the proportion of range currently covered for each species, in order to estimate how far species' met their defined targets (species target achievement), i.e. by dividing this proportion by the defined target.

### (f) Data analyses

We estimated the distinctiveness of species in terms of function and evolutionary history using the fair proportion metric proposed by Isaac *et al.* [14]. We called ED and FD evolutionary and functional distinctiveness, respectively. For each species, this was given by the sum of branch lengths between all nodes from the tip to the root, divided by the number of species subtending each branch. They sum to phylogenetic and functional diversity, that were given by Faith [7] and Petchey & Gaston [53], respectively. For this purpose, we used the function *evol.distinct* from the package *picante* [54] in R [55].

A rooted tree is required when calculating this metric. We ran this function over each of the 100 resolved phylogenetic trees for the four groups. All reported results are the median taken across the 100 phylogenetic trees.

To estimate the effectiveness of conservation in terms of functional and phylogenetic trees, we built on the approach used to estimate the resilience of phylogenetic trees to species extinctions [56,57]. In the extinction risk case, the overall tree of life is scaled by the survival probability of each species. Here, the same reasoning was applied, but the scaling was done using species' target achievements. A conservative approach was taken and it was assumed that the PA system effectively covered a given branch of the phylogenetic or functional tree when at least one of the species subtending to this branch met its conservation target. The proportion of target achievement for internal branches was thus obtained by taking the maximum target achievement among the subtending species.

An interesting feature of this strategy was that the overall diversity of the ‘protected’ tree (i.e. the total sum of branch length including the root [7,53]) could then be compared to the original tree's diversity in order to find the proportion of the tree of life represented within the PA system (‘conservation effectiveness, CE’). CE thus ranges from zero, where all the species are completely outside the PA system, to one, where conservation targets are met for all species and therefore, all branches. We estimated CE for functional and phylogenetic diversity and for each of the four groups. We then compared each result to those obtained from a null model where species’ target achievements were placed at random on the tips of the trees, thus simulating random conservation efforts across species and internal branches.

## 3. Results

### (a) Species coverage within protected area system, target achievement and distinctiveness

**Figure 1.**
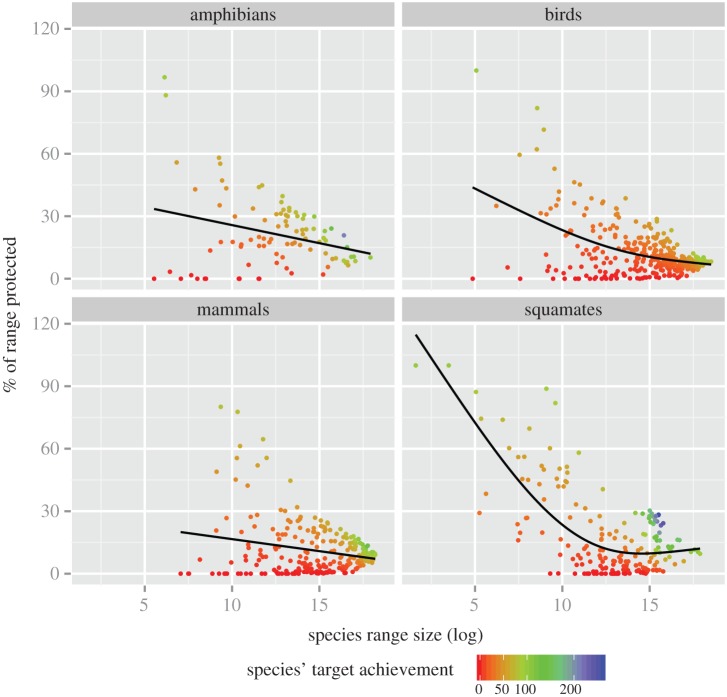
Species range covered by the European PA system according to species range size. Each species is represented by a dot. Species range size is expressed in log scale. Species' target achievement is shown with a colour gradient from red to blue (low to high species target achievement).

Only 8.8% of the European area was represented within the PA system. The proportion of species ranges currently covered varied within and between groups (figure 1). Most importantly, rare species had variously, high and low coverage for amphibians, birds and mammals. The rarest squamates always had a high coverage and relatively high species' target achievement. Conversely, the rarest mammals were generally poorly covered by the European PA system, which meant that they had poor target achievement. Interestingly, some common squamates had species' conservation target achievement much higher than 100% because they have moderate range sizes compared with the other groups that are highly embedded with the PA system.

These results were not influenced by the endemicity status of the species (electronic supplementary material, figure S3). The percentage coverage by PAs for endemic species sometimes reached much higher values than that for the rest of the species, but conversely a relatively high number of endemic species were not well covered by the PA system and did not have high target achievement (electronic supplementary material, figure S4).

In general, species' target achievement was significantly higher for amphibians (median = 56%) than for the three other groups (median = 29%, 26% and 29% for birds, mammals and squamates, respectively; electronic supplementary material, figure S5). In other words, the current PA system achieves better coverage for amphibians than for other tetrapods. These results reflect the level of PA coverage for these species as well as the contrast between Western Europe, which is highly protected, and Eastern Europe, which is poorly protected. Species' target achievement examined in function of species mean position in Europe shows that species whose centre of distribution is in south Eastern Europe (e.g. Anatoly) generally have low species' target achievement, notably for mammals and squamates. Amphibians' distribution, in contrast, is generally centred in southwestern Europe, a region that is well covered by PAs and certainly explains why amphibians have such better coverage (electronic supplementary material, figure S6). For endemics, the median falling within protected zones was 19%, while the median of species' target achievement was 42%.

**Figure 2.**
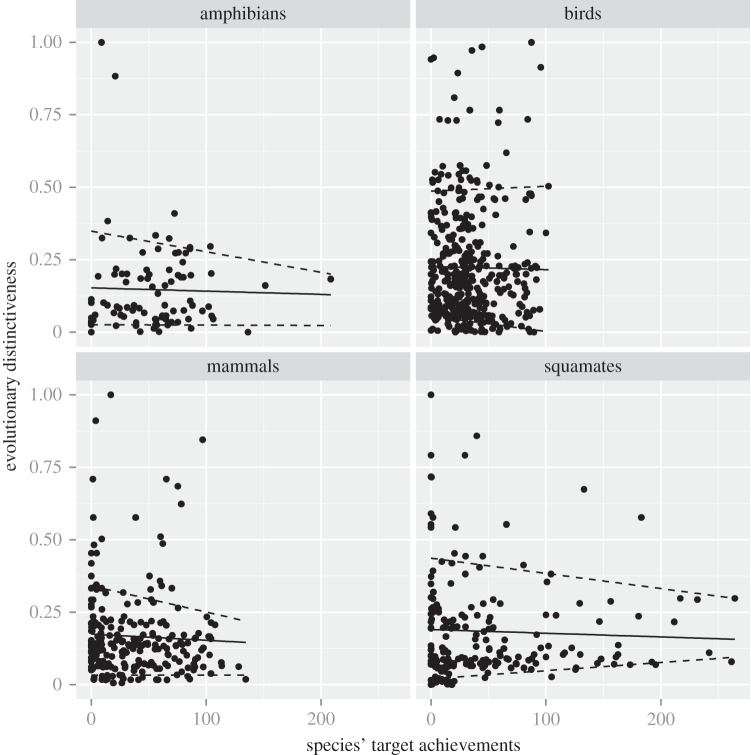
Evolutionary distinctiveness according to species' target achievements. The black line represents the ordinary least square (OLS) regression line, while the upper and lower dashed lines represent the 0.1 and 0.9 quantile regressions. The evolutionary distinctiveness of each species is the median over the 100 maximum-likelihood trees. The black line corresponds to the spline regression between evolutionary distinctiveness and species' target achievement for illustrative purposes.

**Figure 3.**
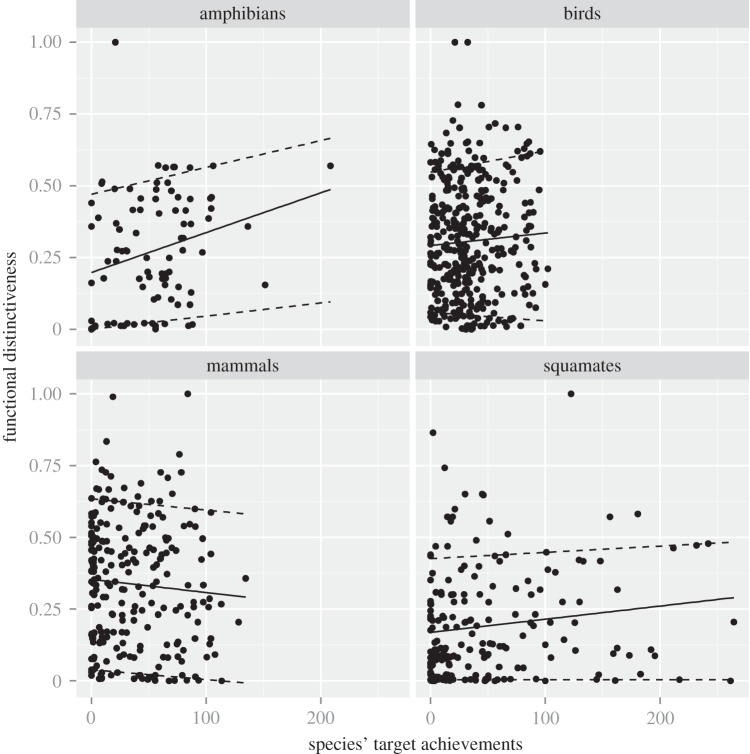
Functional distinctiveness according to species' target achievements. Relationships between species' target achievements and the functional distinctiveness of the four groups. The black line represents the OLS regression line, while the upper and lower dashed lines represent the 0.1 and 0.9 quantile regressions.

In general, weak relationships between species' target achievements and our two measures of distinctiveness (figures 2 and 3) were found. In terms of evolutionary distinctiveness, relationships were relatively weak and non-significant for all species groups, although there was a general negative trend, especially when considering the 90% quantile regression (figure 1; electronic supplementary material, table S1). In other words, the most evolutionary distinct species tended to be less well represented within the current PA system than other species, except for birds. These results held true for endemic species as no significant relationship was found between endemicity status and evolutionary distinctiveness (electronic supplementary material, figure S7).

When focusing on functional distinctiveness, a significant positive relationship was found between species' target achievement and functional distinctiveness for amphibians and squamates only (figure 3; electronic supplementary material, table S1). Functionally distinct amphibian and squamates species tended to be better protected than functionally common species. These results also held true for endemic species as there was no significant relationship between endemicity status and functional distinctiveness (electronic supplementary material, figure S8).

**Figure 4.**
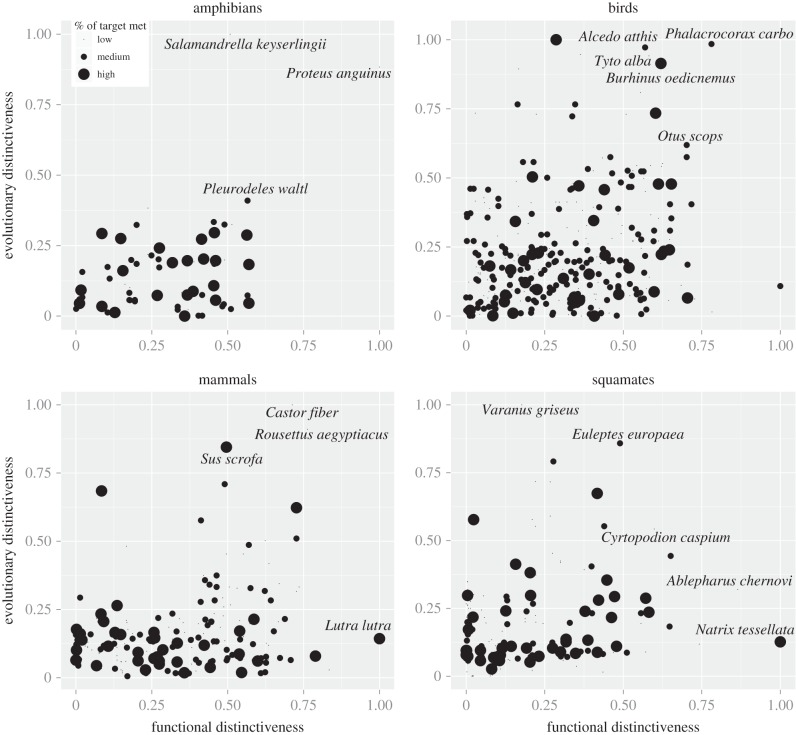
Evolutionary and functional distinctiveness relationship and species' target achievements. Relationships between evolutionary and functional distinctiveness for each species group. Dot size corresponds to species' target achievements (low: less than 25%; high: more than 75%; and medium: between 25 and 75%). The evolutionary distinctiveness of each species is the median over the 100 maximum-likelihood trees.

We then investigated how far species' conservation targets were met along the gradient of evolutionary and functional distinctiveness. The most distinct species in terms of both biodiversity facets were generally poorly protected, except for birds (figure 4). For example, the European beaver *Castor fiber* is one of the most distinct mammals in Europe, both in terms of evolutionary history (it is the only species of the family Castoridae in Europe) and its functions (e.g. as ecosystem engineer), and its target achievement was relatively low. This is probably because most of its range falls within poorly protected eastern parts of Europe. Conversely, the wild boar *Sus scrofa*, a widespread species, which is also relatively distinct both along the evolutionary gradient (it is the only species of the Suidae family in the study area) and the functional gradient, showed a relatively high target achievement, probably owing to its game hunting properties and its high occurrence in well-protected habitats (European mountains are highly protected). Even for amphibians, which generally had higher species' target achievement than the three other groups, the two most distinct species (*Salamandrella keyserlingii* and the olm *Proteus anguinus*) were far from meeting their targets. Although the former species also occurs in eastern Asia, the latter is endemic to Europe and is the only obligate cave-dweller chordate in Europe.

As demonstrated by quantile regressions (figure 2), a trend towards lack of protection for the most evolutionary distinct species of the four groups and the most functionally distinct mammals was observed.

### (b) Conservation effectiveness of evolutionary and functional diversity

**Figure 5.**
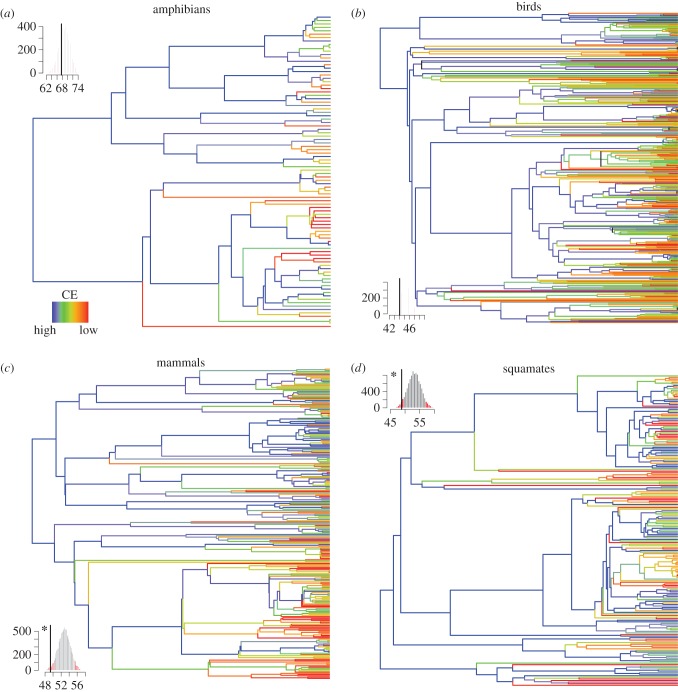
Species' target achievements mapped onto the phylogenetic tree of each group. For the four groups (*a*–*d*), species' target achievements were mapped onto the tree. For each internal branch, the maximum target achievement for the descendant was taken. Colours from red to blue indicate lowest to highest CE. The subplot in the corner represents the CE under random species' target achievements along the tree (9999 repeats). Red indicates the level of significance at 0.05 and 95%. The black line indicates the observed CE. The black asterisk close to the black line indicates significance at 0.05 (one sided). The trees and null models have been carried out over one randomly taken tree from the 100 maximum-likelihood trees for each group.

**Figure 6.**
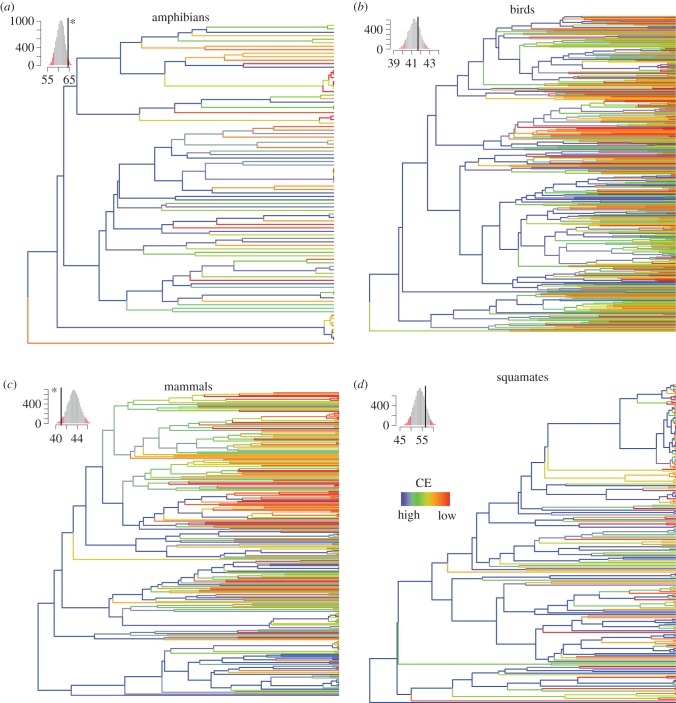
Species' target achievements mapped onto the functional tree of each group. For the four groups, species' target achievements were mapped onto the tree. For each internal branch, the maximum target achievement for the descendant was taken. Colours from red to blue indicate lowest to highest CE. The subplot in the corner represents the CE under random species' target achievements along the tree (9999 repeats). Red indicates the level of significance at 0.05 and 95%. The black line indicates the observed CE. The black asterisk close to the black line indicates significance at 0.05 (one sided). The trees and null models have been carried out over one randomly taken tree from the 100 maximum-likelihood trees for each group.

When scaling up species’ target achievement onto the functional and phylogenetic trees, the CE analysis highlights important differences among groups (table 1, and figures 5 and 6). First of all, CE for amphibians was much higher than for the other groups when both phylogenetic and functional aspects were taken into consideration (table 1). Second, the general trend was for phylogenetic diversity to be less protected than expected given random species’ target achievement across the phylogenetic tree (figure 5). In other words, when looking at the protection of the entire tree of life, evolutionary distinct species tended to have lower target achievement leading to lower CE. This was significant for mammals and squamates but not for amphibians and birds. For squamates, it is likely that the significant effect was partly caused by a strong clustering of low species' target achievement along the phylogeny (electronic supplementary material, figure S9).

**Table 1.** CE in Europe. CE represents the ratio of the represented evolutionary and functional diversity in PAs to the overall evolutionary and functional diversity, respectively (reported in %).

	CE	
	evolutionary history	functional diversity
amphibian	67.7	64.4
birds	44.1	41.7
mammals	49.3	41.0
squamates	48.8	56.5

Third, when considering the overall protection of the functional tree of life, the pattern was reversed with the exception of mammals (figure 6). Functional diversity was overall better protected than expected when compared with random results for squamates, birds and amphibians, although this was significant in the case of amphibians only. Conversely, for mammals, the protection of the functional tree of life was lower than expected when compared with a random distribution of the species' target achievement. Functionally distinct mammals thus tended to have lower target achievements than other mammals. This was also demonstrated by the clustering of low species' target achievements on one part of the functional tree for mammals because of a significant functional signal in species' target achievements (electronic supplementary material, figure S9).

## 4. Discussion

In the current global context of scarce resources allocated to conservation, there is now a general consensus that beyond focusing on the mere number of species or on those with major extinction risks, other facets of biodiversity need to be taken into account [14,16,58,59]. Here, we have pioneered a continental gap analysis by accounting for both functional and phylogenetic components of tetrapod diversity in Europe. We asked whether the current PA system in Europe provides effective coverage for both the evolutionary and functional tetrapod trees of life. We defined those trees and associated measures for distinctiveness for species occurring in the European region under study. These definitions required a large assumption to be made, because distinct species in Europe might not necessarily be distinct at a global scale while rare and unprotected species might actually have large protected ranges outside Europe. For the latter, we argue that as conservation prioritization is often carried out at a continental (i.e. European Union) or at a national level, a precautionary approach is to be recommended [60]. National protection does not necessarily follow global trends but instead may be decided according to interest in a particular species, its rarity and its characteristics. These characteristics, here defined by the contribution of the species to the European phylogenetic and functional trees of life, are crucial for ecosystem functioning as those species might be the ones ensuring long-term stability and resilience [15,16,61]. Our focus on endemic species also supports our strategy since the most evolutionary and functionally distinct species were not necessarily marginal species from outside Europe. Instead, there was no general rule, and the most evolutionary and functionally distinct species could either be endemic to Europe or more cosmopolitan species.

One key result of our analysis was the generally poor conservation target achievement found for all tetrapods except amphibians. There are several explanations for these low values. Our strategy was based on species ranges, which is somewhat arbitrary since it relies on a linear regression between large and small range species along a log scale. Although being generally well accepted [52,62,63], this approach assumes that the overall need for protection linearly scales with the logarithm of range size and that the largest range size may be considered to be the optimal equilibrium range. Furthermore, in our approach, conservation targets are somewhat different among the four clades considered. Average range size of amphibians is much smaller than those of mammals and birds. In practice, restricted birds have range sizes comparable to those of amphibians with relatively broad distributions. As the conservation targets were specifically defined for the four clades, species with similar ranges but belonging to different clades may show different targets. The achievement of targets should be therefore compared among species within given clades, while comparisons between clades should be made with caution. Nevertheless, conservation targets were similar when defined on the basis of IUCN criteria (electronic supplementary information, figure S10), which are not clade specific, thus supporting the robustness of our conclusions. Given that there is no better alternative, as optimal population range sizes are unknown, we can therefore assume that our species-group-based strategy, provides a reasonable estimation of extinction risk at the continental scale [51].

Only 8.8% of Europe is covered by PAs and this coverage is not evenly distributed either in terms of different land cover types (electronic supplementary material, table S2) nor across Europe (electronic supplementary material, figure S1). The level of protection in Europe is highly heterogeneous with a strong western–eastern gradient. The percentage of coverage and associated species' target achievement do reflect this bias (electronic supplementary material, figure S6). The arid steppes of Eastern Europe are covered by almost no PAs. This explains the relatively low species' target achievement for mammals, squamates and, to a lower extent, for birds. Small mammals from the eastern steppic areas (e.g. *Allactaga*, *Sicista*, *Meriones*, *Tatera*, *Rombomys*, *Spalax*, *Mesocricetus* and *Allocricetulus*) have in common to be little covered by the existing network. On the other hand, the representation of land cover type in PAs is biased with respect to European reality. In other words, most PAs, such as national parks, were placed in remote areas or where it was convenient and were not necessarily designed only in terms of specific conservation purposes. This is not true for Natura 2000, which was specifically designed to protect specific habitats or species. This bias towards failing to represent different land cover types in the PA network has obvious consequences on species protection and therefore on species' target achievement. For instance, the most well-represented land cover types in PAs are permanent snow, bare areas and salt hardpans, while croplands, grasslands, mosaic vegetation and aquatic habitats are obviously among the least represented within PAs (2–4%, electronic supplementary material, table S2). Although the global intensification of agriculture and ever-increasing urbanization are known to favour local extinctions [64], some of these areas are also known to be settings that harbour specific types of biodiversity, notably areas of low intensity and low input farming systems in Europe (i.e. concept of High Nature Value [65]). The same applies to aquatic habitats that are poorly protected but so important for lots of birds and mammal species. This is perhaps why mammals and birds seem to have low target achievement. In Europe, many bird and mammal species are associated with traditional agricultural landscapes: agricultural intensification and the abandonment of traditional agricultural practices are causing a widespread decline in species [66]. Traditional conservation approaches, such as PAs, may therefore not be the most appropriate tools for the conservation of farmland birds, and other approaches such agri-environmental schemes need to be implemented for their conservation [67,68]. However, these schemes are not absolutely effective, and it is impossible to obtain comprehensive and broad-scale information about them [66]. For these reasons we did not integrate agri-environmental schemes into our analyses, and conservation actions taken to protect farmland birds may be underestimated. Nevertheless, farmland bird communities carry out key functions in ecosystems, such as the regulation of pest insects [69], and constitute a major priority for biodiversity conservation in Europe.

Interestingly, amphibians emerged as the best-protected species in Europe within the scope of our species targets. Amphibians are the vertebrates with the smallest ranges and highest endemism in Europe. Several of the endemic amphibians with the smallest ranges (e.g. *Hydromantes* cave salamanders, *Calotriton arnoldi*, *Salamandra lanzai*) are concentrated on islands and mountainous ranges of Southern Europe (e.g. Sardinia, the East of the Iberian Peninsula, the southwestern Alps [70]; electronic supplementary material, figure S6). In these areas, the preservation of highly endemic and endangered amphibians attracts appropriate conservation efforts. For instance, the protection of endemic amphibians had a major role in the creation of several Natura 2000 areas in Sardinia. Our analyses show that this system seems to be relatively effective, although it may be jeopardized later by climate change [23]. Nevertheless, the level of protection remains poor for some restricted and poorly known species endemic to Anatolia, such as *Mertensiella caucasica* and basically the whole genus *Lyciasalamandra* (with the exception of only *Lyciasalamandra helverseni*, which reached slightly more than 50% of its target) and *Neurergus*. Notably, all species of the genus *Lyciasalamandra* are endemic to small areas of Turkey, with a couple of species expanding their range towards Greece. They are threatened according to IUCN categories (www.iucnredlist.org) and subject to huge problems linked to possible future tourism developments (with the associated habitat loss) and over-collection for scientific purposes (electronic supplementary material, figure S6). The same problem is also faced by the two species in the genus *Neurergus*, one of which (*Neurergus strauchii*) is endemic to Turkey, both of which have very low coverage by PAs.

When phylogenetic trees of life and the scaling up of species coverage along the branches is examined, the most evolutionary distinct species seem to be less protected than expected when compared with random results or there appears to be strong phylogenetic clustering of species with low species' target achievement, in particular for squamates. This is important since it has been shown here that these evolutionary distinct species are not necessarily marginal species in Europe but instead are often even endemic to Europe. The loss of evolutionary distinct species could thus affect the European tree of life disproportionally, and thereby have a tremendous overall effect on the feature diversity they represent [71]. Recently, Mouillot *et al.* [16] demonstrated that rare species usually bear distinct functions that could put ecosystems functioning at risk if they go extinct, therefore urging us to consider functional distinctiveness in conservation assessments. This has been done here and it has been shown that target achievement for one of the most functionally distinct species, the olm, is far from being met. However, apart from in the case of mammals, our assessment demonstrates that the functional tree of life is better protected than the evolutionary tree of life (figure 5) and that the contribution of distinct species to its protection was significant. The choice of traits and the functions these traits capture obviously defines this assessment. Functional traits chosen here include behavioural traits during feeding to reflect how species acquire resources from their environment (feeding behaviour and activity), and log-transformed body mass/length and diet traits are used to reflect the resource requirements. These traits determine the impact of a given organism on community structure and ecosystem functioning [72,73], although the distinction between effect and response traits (traits that stand for the response of organisms to environmental change) is not always straightforward for animals [42]. The set of traits selected, can therefore be expected to be an appropriate proxy of functions, such as plant population regulation and seed transportation, which thereby help to maintain plant diversity by lowering the effects of interspecific competition,and enhancing dispersal [74,75]. As another example, cavity-drillers and nest-burrowers are recognized as ecosystem engineers that provide shelter to additional species [43,75,76], whrease large mammals and top carnivores are known to have a disproportionate role in the regulation of the whole food chain [77]. Besides these functions that are essential to the functioning of the ecosystem, many tetrapods also provide important educational, cultural and recreational services for nature enthusiasts and contribute to global nutrient dynamics [75]. It is therefore important that these functions are adequately protected. Thus, the results of this study argue for the incorporation of both those aspects, evolutionary history and functionality, in conservation planning. Next steps should include assessment of how the current European PA system could be extended to Eastern Europe at minimal cost but taking tetrapod evolutionary history into account, and maximizing the range of fundamental and derived ecosystem services those species could sustain, to generate a win–win situation. Additionally, the dual effects of climate and land-use change on the phylogenetic and functional diversity of Europe should be included in future conservation planning as these changes could jeopardize the effectiveness of the current protection system [23,57,78]. The spatial data used here could be very useful in drawing up the borders for future long-term planning for phylogenetic and functional diversity conservation at a European level.

## Supplementary Material

Supplementary material
