# Supplementary material for: Conserving the functional and phylogenetic trees of life of European tetrapods
Source: Philos Trans R Soc Lond B Biol Sci. 2015 Feb 19;370(1662):20140005. doi: 10.1098/rstb.2014.0005 (PMC4290419; doi:10.1098/rstb.2014.0005)
Supplement: Supplementary material [file rstb20140005supp1.pdf]

Phil. Trans. R. Soc. B. doi : 10.1098/rstb.2014.0005

Electronic Supplementary Materials

Conserving the functional and phylogenetic trees of life of European tetrapods

Wilfried Thuiller[1,2], Luigi Maiorano[2], Florent Mazel[1,2], François Guilhaumon[3], Gentile Francesco Ficetola[1,2], Sébastien Lavergne[1,2], Julien Renaud[1,2], Cristina Roquet[1,2] and David Mouillot[3].

[1] Univ. Grenoble Alpes, LECA, F-38000 Grenoble, France

[2] CNRS, LECA, F-38000 Grenoble, France

[3] Dipartimento di Biologia e Biotecnologie "Charles Darwin", Università di Roma "La Sapienza" 00185 Roma, Italy

[4] Laboratoire ECOSYM, UMR 5119 CNRS-UM2-IRD-IFREMER, Place Eugène Bataillon cc 93, 34095 Montpellier, France

Subject area: ecology, environmental science,

Corresponding author: Wilfried Thuiller, wilfried.thuiller@ujf-grenoble.fr

Species list

Amphibians	Birds	Mammals	Squamates
Alytes cisternasii	*Accipiter brevipes*	*Acomys minous*	*Ablepharus budaki*
Alytes dickhilleni	*Accipiter gentilis*	*Acomys nesiotes*	*Ablepharus chernovi*
Alytes muletensis	*Accipiter nisus*	*Alces alces*	*Ablepharus kitaibelii*
Alytes obstetricans	*Acrocephalus agricola*	*Allactaga elater*	*Acanthodactylus boskianus*
Bombina bombina	*Acrocephalus arundinaceus*	*Allactaga euphratica*	*Acanthodactylus erythrurus*
Bombina pachypus	*Acrocephalus dumetorum*	*Allactaga major*	*Acanthodactylus grandis*
Bombina variegata	*Acrocephalus melanopogon*	*Allactaga sibirica*	*Acanthodactylus schreiberi*
Bufo balearicus	*Acrocephalus paludicola*	*Allactaga williamsi*	*Algyroides fitzingeri*
Bufo boulengeri	*Acrocephalus palustris*	*Allocricetulus eversmanni*	*Algyroides marchi*
Bufo bufo	*Acrocephalus schoenobaenus*	*Apodemus agrarius*	*Algyroides moreoticus*
Bufo calamita	*Acrocephalus scirpaceus*	*Apodemus alpicola*	*Algyroides nigropunctatus*
Bufo siculus	*Actitis hypoleucos*	*Apodemus flavicollis*	*Alsophylax pipiens*
Bufo variabilis	*Aegithalos caudatus*	*Apodemus hyrcanicus*	*Anatololacerta anatolica*
Bufo verrucosissimus	*Aegolius funereus*	*Apodemus mystacinus*	*Anatololacerta danfordi*
Bufo viridis	*Aegypius monachus*	*Apodemus ponticus*	*Anatololacerta oertzeni*
Calotriton arnoldi	*Alauda arvensis*	*Apodemus sylvaticus*	*Anguis cephallonica*
Calotriton asper	*Alcedo atthis*	*Apodemus uralensis*	*Anguis fragilis*
Chioglossa lusitanica	*Alectoris barbara*	*Apodemus witherbyi*	*Apathya cappadocica*
Discoglossus galganoi	*Alectoris chukar*	*Arvicola amphibius*	*Archaeolacerta bedriagae*
Discoglossus jeanneae	*Alectoris graeca*	*Arvicola sapidus*	*Asaccus elisae*
Discoglossus montalentii	*Alectoris rufa*	*Arvicola scherman*	*Blanus cinereus*
Discoglossus pictus	*Anas acuta*	*Atelerix algirus*	*Blanus strauchi*
Discoglossus sardus	*Anas clypeata*	*Barbastella barbastellus*	*Chalcides bedriagai*
Euproctus montanus	*Anas crecca*	*Barbastella leucomelas*	*Chalcides bistriatus*
Euproctus platycephalus	*Anas platyrhynchos*	*Bison bonasus*	*Chalcides chalcides*
Hydromantes ambrosii	*Anas querquedula*	*Calomyscus urartensis*	*Chalcides ocellatus*
Hydromantes flavus	*Anser anser*	*Canis aureus*	*Chalcides sexlineatus*
Hydromantes genei	*Anser brachyrhynchus*	*Canis lupus*	*Chalcides simonyi*
Hydromantes imperialis	*Anser erythropus*	*Capra caucasica*	*Chalcides striatus*
Hydromantes italicus	*Anser fabalis*	*Capra ibex*	*Chalcides viridanus*
Hydromantes sarrabusensis	*Anthropoides virgo*	*Capra pyrenaica*	*Chamaeleo africanus*
Hydromantes strinatii	*Anthus berthelotii*	*Capreolus capreolus*	*Chamaeleo chamaeleon*
Hydromantes supramontis	*Anthus campestris*	*Capreolus pygargus*	*Coronella austriaca*
Hyla arborea	*Anthus cervinus*	*Castor fiber*	*Coronella girondica*
Hyla intermedia	*Anthus petrosus*	*Cervus elaphus*	*Cyrtopodion caspium*
Hyla meridionalis	*Anthus pratensis*	*Chionomys gud*	*Cyrtopodion heterocercum*
Hyla sarda	*Anthus spinoletta*	*Chionomys nivalis*	*Cyrtopodion kotschyi*
Hyla savignyi	*Anthus trivialis*	*Chionomys roberti*	*Cyrtopodion scabrum*
Lissotriton boscai	*Apus apus*	*Cricetulus migratorius*	*Dalmatolacerta oxycephala*
Lissotriton helveticus	*Apus caffer*	*Cricetus cricetus*	*Darevskia armeniaca*
Lissotriton italicus	*Apus melba*	*Crocidura canariensis*	*Darevskia bendimahiensis*
Lissotriton montandoni	*Apus pallidus*	*Crocidura leucodon*	*Darevskia clarkorum*
Lissotriton vulgaris	*Aquila chrysaetos*	*Crocidura russula*	*Darevskia dahli*
Lyciasalamandra antalyana	*Aquila clanga*	*Crocidura sicula*	*Darevskia derjugini*
Lyciasalamandra atifi	*Aquila heliaca*	*Crocidura suaveolens*	*Darevskia mixta*
Lyciasalamandra billae	*Aquila pomarina*	*Dama dama*	*Darevskia nairensis*
Lyciasalamandra fazilae	*Ardea cinerea*	*Desmana moschata*	*Darevskia parvula*
Lyciasalamandra flavimembris	*Ardea purpurea*	*Dicrostonyx torquatus*	*Darevskia portschinskii*
Lyciasalamandra helverseni	*Ardeola ralloides*	*Dinaromys bogdanovi*	*Darevskia praticola*
Lyciasalamandra luschani	*Arenaria interpres*	*Diplomesodon pulchellum*	*Darevskia raddei*
Mertensiella caucasica	*Asio flammeus*	*Dipus sagitta*	*Darevskia rostombekovi*
Mesotriton alpestris	*Asio otus*	*Dryomys laniger*	*Darevskia rudis*
Neurergus crocatus	*Athene noctua*	*Dryomys nitedula*	*Darevskia sapphirina*
Neurergus strauchii	*Aythya ferina*	*Eliomys melanurus*	*Darevskia unisexualis*
Ommatotriton ophryticus	*Aythya fuligula*	*Eliomys quercinus*	*Darevskia uzzelli*
Ommatotriton vittatus	*Aythya marila*	*Ellobius lutescens*	*Darevskia valentini*
Pelobates cultripes	*Aythya nyroca*	*Ellobius talpinus*	*Dinarolacerta montenegrina*
Pelobates fuscus	*Bombycilla garrulus*	*Eptesicus bottae*	*Dinarolacerta mosorensis*
Pelobates syriacus	*Bonasa bonasia*	*Eptesicus serotinus*	*Dolichophis caspius*
Pelodytes caucasicus	*Botaurus stellaris*	*Erinaceus concolor*	*Dolichophis cypriensis*
Pelodytes ibericus	*Branta leucopsis*	*Erinaceus europaeus*	*Dolichophis jugularis*
Pelodytes punctatus	*Bubo bubo*	*Erinaceus roumanicus*	*Dolichophis schmidti*
Pleurodeles waltl	*Bubulcus ibis*	*Felis chaus*	*Eirenis decemlineatus*
Proteus anguinus	*Bucanetes githagineus*	*Felis margarita*	*Eirenis levantinus*
Rana arvalis	*Bucephala clangula*	*Felis silvestris*	*Eirenis lineomaculatus*
Rana bergeri	*Burhinus oedicnemus*	*Galemys pyrenaicus*	*Eirenis modestus*
Rana dalmatina	*Buteo buteo*	*Gazella subgutturosa*	*Eirenis punctatolineatus*
Rana esculenta	*Buteo lagopus*	*Genetta genetta*	*Eirenis rothii*
Rana graeca	*Buteo rufinus*	*Glis glis*	*Elaphe dione*
Rana iberica	*Calandrella brachydactyla*	*Gulo gulo*	*Elaphe quatuorlineata*

Rana italica
Rana kurtmuelleri
Rana latastei
Rana lessonae
Rana perezi
Rana pyrenaica
Rana ridibunda
Rana temporaria
Salamandra atra
Salamandra corsica
Salamandra infraimmaculata
Salamandra lanzai
Salamandra salamandra
Salamandrella keyserlingii
Salamandrina perspicillata
Salamandrina terdigitata
Triturus carnifex
Triturus cristatus
Triturus dobrogicus
Triturus karelinii
Triturus marmoratus
Triturus pygmaeus

Calandrella cheleensis
Calandrella rufescens
Calcarius lapponicus
Calidris alpina
Calidris maritima
Calidris minuta
Calidris temminckii
Caprimulgus europaeus
Caprimulgus ruficollis
Carduelis cannabina
Carduelis carduelis
Carduelis chloris
Carduelis flammea
Carduelis flavirostris
Carduelis hornemanni
Carduelis spinus
Carpodacus erythrinus
Cercotrichas galactotes
Certhia brachydactyla
Certhia familiaris
Cettia cetti
Charadrius alexandrinus
Charadrius asiaticus
Charadrius dubius
Charadrius hiaticula
Charadrius morinellus
Chersophilus duponti
Chlidonias hybridus
Chlidonias leucopterus
Chlidonias niger
Ciconia ciconia
Ciconia nigra
Cinclus cinclus
Circaetus gallicus
Circus aeruginosus
Circus cyaneus
Circus macrourus
Circus pygargus
Cisticola juncidis
Clamator glandarius
Clangula hyemalis
Coccothraustes coccothraustes
Columba livia
Columba oenas
Columba palumbus
Coracias garrulus
Corvus corax
Corvus corone
Corvus frugilegus
Corvus monedula
Coturnix coturnix
Crex crex
Cuculus canorus
Cyanopica cyana (cyanus)
Cygnus cygnus
Cygnus olor
Delichon urbica
Dendrocopos major
Dendrocopos medius
Dendrocopos minor
Dryocopus martius
Egretta garzetta
Elanus caeruleus
Emberiza aureola
Emberiza caesia
Emberiza cia
Emberiza cirlus
Emberiza citrinella
Emberiza hortulana
Emberiza melanocephala
Emberiza pusilla
Emberiza rustica
Emberiza schoeniclus
Eremophila alpestris
Erithacus rubecula
Falco biarmicus

Hemiechinus auritus
Herpestes ichneumon
Hyaena hyaena
Hystrix cristata
Hystrix indica
Lagurus lagurus
Lemmus lemmus
Lepus capensis
Lepus castroviejoi
Lepus corsicanus
Lepus europaeus
Lepus granatensis
Lepus timidus
Lepus tolai
Lutra lutra
Lynx lynx
Lynx pardinus
Marmota baibacina
Marmota marmota
Martes foina
Martes martes
Martes zibellina
Meles leucurus
Meles meles
Meriones crassus
Meriones dahli
Meriones libycus
Meriones meridianus
Meriones persicus
Meriones tamariscinus
Meriones vinogradovi
Mesocricetus auratus
Mesocricetus brandti
Mesocricetus newtoni
Mesocricetus raddei
Micromys minutus
Microtus agrestis
Microtus arvalis
Microtus brachycercus
Microtus cabrerae
Microtus daghestanicus
Microtus duodecimcostatus
Microtus gerbei
Microtus gregalis
Microtus guentheri
Microtus levis
Microtus lusitanicus
Microtus majori
Microtus middendorffii
Microtus oeconomus
Microtus savii
Microtus socialis
Microtus subterraneus
Microtus tatricus
Mus macedonicus
Mus musculus
Mus spicilegus
Mus spretus
Muscardinus avellanarius
Mustela erminea
Mustela eversmanii
Mustela lutreola
Mustela nivalis
Mustela putorius
Mustela sibirica
Myodes glareolus
Myodes rufocanus
Myodes rutilus
Myomimus roachi
Myopus schisticolor
Myotis bechsteinii
Myotis blythii
Myotis brandtii
Myotis capaccinii
Myotis dasycneme
Myotis daubentonii

Elaphe sauromates
Eremias arguta
Eremias persica
Eremias pleskei
Eremias strauchi
Eryx jaculus
Euleptes europaea
Eumeces schneideri
Gallotia atlantica
Gallotia bravoana
Gallotia caesaris
Gallotia galloti
Gallotia intermedia
Gallotia simonyi
Gallotia stehlini
Hellenolacerta graeca
Hemidactylus turcicus
Hemorrhois algirus
Hemorrhois hippocrepis
Hemorrhois nummifer
Hemorrhois ravergieri
Hierophis gemonensis
Hierophis viridiflavus
Iberolacerta aranica
Iberolacerta aurelioi
Iberolacerta bonnali
Iberolacerta cyreni
Iberolacerta galani
Iberolacerta horvathi
Iberolacerta martinezricai
Iberolacerta monticola
Iranolacerta brandtii
Lacerta agilis
Lacerta bilineata
Lacerta media
Lacerta schreiberi
Lacerta strigata
Lacerta trilineata
Lacerta viridis
Laudakia caucasia
Laudakia stellio
Leptotyphlops macrorhynchus
Macroprotodon brevis
Macroprotodon cucullatus
Macroprotodon mauritanicus
Macrovipera lebetina
Macrovipera schweizeri
Malpolon monspessulanus
Mesalina brevirostris
Montivipera raddei
Montivipera wagneri
Montivipera xanthina
Natrix maura
Natrix natrix
Natrix tessellata
Ophiomorus punctatissimus
Ophisops elegans
Parvilacerta parva
Phoenicolacerta laevis
Phoenicolacerta troodica
Phrynocephalus helioscopus
Phrynocephalus mystaceus
Phrynocephalus persicus
Platyceps collaris
Platyceps najadum
Platyceps ventromaculatus
Podarcis atrata
Podarcis bocagei
Podarcis carbonelli
Podarcis erhardii
Podarcis filfolensis
Podarcis gaigeae
Podarcis hispanica
Podarcis lilfordi
Podarcis melisellensis
Podarcis milensis

Falco cherrug
Falco columbarius
Falco eleonorae
Falco naumanni
Falco peregrinus
Falco rusticolus
Falco subbuteo
Falco tinnunculus
Falco vespertinus
Ficedula albicollis
Ficedula hypoleuca
Ficedula parva
Ficedula semitorquata
Fringilla coelebs
Fringilla montifringilla
Fulica atra
Fulica cristata
Galerida cristata
Galerida theklae
Gallinago gallinago
Gallinago media
Gallinula chloropus
Garrulus glandarius
Gavia arctica
Gavia immer
Gavia stellata
Gelochelidon nilotica
Glareola nordmanni
Glareola pratincola
Glaucidium passerinum
Grus grus
Gypaetus barbatus
Gyps fulvus
Haematopus ostralegus
Haliaeetus albicilla
Hieraaetus fasciatus
Hieraaetus pennatus
Himantopus himantopus
Hippolais icterina
Hippolais olivetorum
Hippolais pallida
Hippolais polyglotta
Hirundo daurica
Hirundo rustica
Ixobrychus minutus
Jynx torquilla
Lagopus lagopus
Lagopus mutus
Lanius collurio
Lanius excubitor
Lanius minor
Lanius nubicus
Lanius senator
Larus argentatus
Larus audouinii
Larus canus
Larus fuscus
Larus hyperboreus
Larus ichthyaetus
Larus marinus
Larus melanocephalus
Larus minutus
Larus ridibundus
Limicola falcinellus
Limosa lapponica
Limosa limosa
Locustella fluviatilis
Locustella luscinioides
Locustella naevia
Loxia curvirostra
Loxia pytyopsittacus
Loxia scotica
Lullula arborea
Luscinia luscinia
Luscinia megarhynchos
Luscinia svecica

Myotis emarginatus
Myotis myotis
Myotis mystacinus
Myotis nattereri
Myotis nipalensis
Myotis punicus
Neomys anomalus
Neomys fodiens
Neomys teres
Nyctalus azoreum
Nyctalus lasiopterus
Nyctalus leisleri
Nyctalus noctula
Ochotona hyperborea
Ochotona pusilla
Oryctolagus cuniculus
Ovis aries
Panthera pardus
Pipistrellus kuhlii
Pipistrellus maderensis
Pipistrellus nathusii
Pipistrellus pipistrellus
Pipistrellus pygmaeus
Plecotus auritus
Plecotus austriacus
Plecotus kolombatovici
Plecotus teneriffae
Prometheomys schaposchnikowi
Pteromys volans
Pygeretmus platyurus
Pygeretmus pumilio
Rangifer tarandus
Rhinolophus blasii
Rhinolophus euryale
Rhinolophus ferrumequinum
Rhinolophus hipposideros
Rhinolophus mehelyi
Rhombomys opimus
Rousettus aegyptiacus
Rupicapra pyrenaica
Rupicapra rupicapra
Saiga tatarica
Sciurus anomalus
Sciurus vulgaris
Sicista armenica
Sicista betulina
Sicista caucasica
Sicista kazbegica
Sicista kluchorica
Sicista severtzovi
Sicista strandi
Sicista subtilis
Sorex alpinus
Sorex antinorii
Sorex araneus
Sorex caecutiens
Sorex coronatus
Sorex granarius
Sorex isodon
Sorex minutissimus
Sorex minutus
Sorex raddei
Sorex samniticus
Sorex satunini
Sorex tundrensis
Sorex volnuchini
Spalax arenarius
Spalax ehrenbergi
Spalax giganteus
Spalax graecus
Spalax leucodon
Spalax nehringi
Spalax uralensis
Spalax zemni
Spermophilus citellus
Spermophilus fulvus

Podarcis muralis
Podarcis peloponnesiaca
Podarcis pityusensis
Podarcis raffonei
Podarcis sicula
Podarcis taurica
Podarcis tiliguerta
Podarcis wagleriana
Psammodromus algirus
Psammodromus hispanicus
Pseudocerastes persicus
Pseudopus apodus
Rhinechis scalaris
Spalerosophis diadema
Stenodactylus grandiceps
Tarentola angustimentalis
Tarentola bischoffi
Tarentola boettgeri
Tarentola delalandii
Tarentola gomerensis
Tarentola mauritanica
Teira dugesii
Telescopus fallax
Timon lepidus
Timon princeps
Trachylepis aurata
Trachylepis septemtaeniata
Trachylepis vittata
Trapelus agilis
Typhlops vermicularis
Varanus griseus
Vipera ammodytes
Vipera aspis
Vipera berus
Vipera dinniki
Vipera eriwanensis
Vipera kaznakovi
Vipera latastei
Vipera lotievi
Vipera nikolskii
Vipera renardi
Vipera seoanei
Vipera transcaucasiana
Vipera ursinii
Zamenis hohenackeri
Zamenis lineatus
Zamenis longissimus
Zamenis persica
Zamenis situla
Zootoca vivipara

	Lymnocryptes minimus<br>*Marmaronetta angustirostris*<br>*Melanitta fusca*<br>*Melanitta nigra*<br>*Melanocorypha calandra*<br>*Mergus merganser*<br>*Mergus serrator*<br>*Merops apiaster*<br>*Miliaria calandra*<br>*Milvus migrans*<br>*Milvus milvus*<br>*Monticola saxatilis*<br>*Monticola solitarius*<br>*Motacilla alba*<br>*Motacilla cinerea*<br>*Motacilla citreola*<br>*Motacilla flava*<br>*Muscicapa striata*<br>*Neophron percnopterus*<br>*Netta rufina*<br>*Nucifraga caryocatactes*<br>*Numenius arquata*<br>*Numenius phaeopus*<br>*Nyctea scandiaca*<br>*Nycticorax nycticorax*<br>*Oenanthe hispanica*<br>*Oenanthe isabellina*<br>*Oenanthe oenanthe*<br>*Oenanthe pleschanka*<br>*Oriolus oriolus*<br>*Otis tarda*<br>*Otus scops*<br>*Oxyura leucocephala*<br>*Pandion haliaetus*<br>*Panurus biarmicus*<br>*Parus ater*<br>*Parus caeruleus*<br>*Parus cinctus*<br>*Parus cristatus*<br>*Parus cyanus*<br>*Parus lugubris*<br>*Parus major*<br>*Parus montanus*<br>*Parus palustris*<br>*Passer domesticus*<br>*Passer hispaniolensis*<br>*Passer montanus*<br>*Perdix perdix*<br>*Perisoreus infaustus*<br>*Pernis apivorus*<br>*Petronia petronia*<br>*Phalacrocorax carbo*<br>*Phalaropus lobatus*<br>*Phasianus colchicus*<br>*Philomachus pugnax*<br>*Phoenicurus ochruros*<br>*Phoenicurus phoenicurus*<br>*Phylloscopus bonelli*<br>*Phylloscopus borealis*<br>*Phylloscopus sibilatrix*<br>*Phylloscopus trochiloides*<br>*Phylloscopus trochilus*<br>*Pica pica*<br>*Picoides tridactylus*<br>*Picus canus*<br>*Picus viridis*<br>*Pinicola enucleator*<br>*Platalea leucorodia*<br>*Plectrophenax nivalis*<br>*Plegadis falcinellus*<br>*Pluvialis apricaria*<br>*Pluvialis squatarola*<br>*Podiceps auritus*<br>*Podiceps cristatus*<br>*Podiceps grisegena*<br>*Podiceps nigricollis*	*Spermophilus major*<br>*Spermophilus musicus*<br>*Spermophilus pygmaeus*<br>*Spermophilus suslicus*<br>*Spermophilus xanthoprymnus*<br>*Suncus etruscus*<br>*Sus scrofa*<br>*Tadarida teniotis*<br>*Talpa caeca*<br>*Talpa caucasica*<br>*Talpa davidiana*<br>*Talpa europaea*<br>*Talpa levantis*<br>*Talpa occidentalis*<br>*Talpa romana*<br>*Talpa stankovici*<br>*Tamias sibiricus*<br>*Tatera indica*<br>*Ursus arctos*<br>*Ursus maritimus*<br>*Vespertilio murinus*<br>*Vormela peregusna*<br>*Vulpes corsac*<br>*Vulpes vulpes*	

	Porphyrio porphyrio			
	Porzana parva			
	Porzana porzana			
	Porzana pusilla			
	Prunella collaris			
	Prunella modularis			
	Pterocles alchata			
	Pterocles orientalis			
	Ptyonoprogne rupestris			
	Pyrrhocorax graculus			
	Pyrrhocorax pyrrhocorax			
	Pyrrhula pyrrhula			
	Rallus aquaticus			
	Recurvirostra avosetta			
	Regulus ignicapillus			
	Regulus regulus			
	Remiz pendulinus			
	Riparia riparia			
	Rissa tridactyla			
	Saxicola rubetra			
	Saxicola torquata			
	Scolopax rusticola			
	Serinus citrinella			
	Serinus serinus			
	Sitta europaea			
	Sitta krueperi			
	Sitta neumayer			
	Somateria mollissima			
	Stercorarius longicaudus			
	Stercorarius parasiticus			
	Stercorarius pomarinus			
	Stercorarius skua			
	Sterna albifrons			
	Sterna caspia			
	Sterna dougallii			
	Sterna hirundo			
	Sterna paradisaea			
	Sterna sandvicensis			
	Streptopelia decaocto			
	Streptopelia turtur			
	Strix aluco			
	Strix nebulosa			
	Strix uralensis			
	Sturnus roseus			
	Sturnus unicolor			
	Sturnus vulgaris			
	Surnia ulula			
	Sylvia atricapilla			
	Sylvia borin			
	Sylvia cantillans			
	Sylvia communis			
	Sylvia conspicillata			
	Sylvia curruca			
	Sylvia hortensis			
	Sylvia melanocephala			
	Sylvia rueppelli			
	Sylvia sarda			
	Sylvia undata			
	Tachybaptus ruficollis			
	Tadorna ferruginea			
	Tadorna tadorna			
	Tetrao tetrix			
	Tetrao urogallus			
	Tetrax tetrax			
	Tichodroma muraria			
	Tringa erythropus			
	Tringa glareola			
	Tringa nebularia			
	Tringa ochropus			
	Tringa stagnatilis			
	Tringa totanus			
	Troglodytes troglodytes			
	Turdus iliacus			
	Turdus merula			
	Turdus philomelos			
	Turdus pilaris			

	Turdus torquatus<br>*Turdus viscivorus*<br>*Tyto alba*<br>*Upupa epops*<br>*Vanellus gregarius*<br>*Vanellus vanellus*<br>*Xenus cinereus*		

Functional trait database.

To make sure all analyses were comparable throughout the four groups, we selected similar or equivalent traits for the four groups. For instance, body mass was used for both birds and mammals while body length was used for amphibians and squamates.
We summarised below the different classes used for each group and for each traits and their meaning. We also added the list of publications where the data where gathered. For birds, most data were extracted from Pearman *et al.* [1].

Diet traits.

- **Adult:** Mushrooms, mosses/lichens, seeds/nuts/grains, fruits/berries, vegetarian, invertebrates, fishes, small mammals, large mammals, herptiles, bird eggs, small birds, large birds, bones, carrion, coprofagus.
- **Larvae-juveniles (amphibians):** vegetarian, invertebrates, carrion, detritus

Adult feeding behaviours

- **Adult:** Opportunistic, hunting, browser, grazer. For birds: pursuit/air/aquatic, sally, foliage gleaning, pounce, grazing, pick/peck/stab, dig, overturning objects, probe and filter.
- **Larvae-juveniles (amphibians):** Opportunistic, hunting.

Reproduction site

- ground, artificial/building, elevated (tree/shrubs), underground water, cave/fissures/borrows, lodge, temporary water, brooks/springs/small rivers, rivers, puddles/ponds/pools/small lakes, lakes, brackish waters, sea.

Activity

- Nortural, crepuscular, diurnal and arithmic.

Nesting location

- viviparous, ovoviviparous, elevated, tree hole/fissure in the bark, ground, rocks, building/artificial, underground water, cave/fissures/borrows, lodge, temporary water, brooks/springs/small rivers, puddles/ponds/pools/small lakes, brackish waters, parthenogenetic.

Additional references

Amphibians

Baier F, Sparrow DJ, Wiedl HJ. 2013. The amphibians and reptiles of Cyprus. Chimaira Editions, Frankfurt, 362 pp.

Gasc JP, Cabela A, Crnobrnja-Isailovic J, Dolmen D, Grossenbacher K, Haffner P, Lescure J, Martens H, Martínez Rica JP, Maurin H, Oliveira ME, Sofianidou TS, Veith M, Zuiderwijk A (Eds). 1997. Atlas of amphibians and reptiles in Europe. Collection Patrimoines Naturels, 29, Societas Europaea Herpetologica, Muséum National d'Histoire Naturelle & Service du Patrimoine Naturel, Paris, 496 pp.

Golemanski V et al. 2007. Red data book of the Republic of Bulgaria. Volume 2 – Animals. http://e-ecodb.bas.bg/rdb/en/vol2/

Lanza B, Andreone F, Bologna MA, Corti C, Razzetti E. (Eds). 2007. Fauna d'Italia Vol 42 – Amphibia. Calderini, Bologna.

Segev O. 2009. Conservation and ecology of the endangered fire salamander (*Salamandra infraimmaculata*). PhD Dissertation. University of Haifa

Sindaco R, Doria G, Razzetti E, Bernini F (Eds). 2006. Atlas of Italian amphibians and reptiles. Societas Herpetologica Italica, Edizioni Polistampa, Firenze, 792 pp.

Valakos ED, Pafilis P, Sotiropoulos K, Lymberakis P, Maragou P, Foufopulos J. 2008. The amphibians and reptiles of Greece. Chimaira Editions, Frankfurt, 563 pp.

Web Sites

www.herpfrance.com

www.arkive.org

www.edgeofexistence.org/amphibians/

www.iucnredlist.org

http://eol.org

www.vertebradosiberico.org

http://www.demogr.mpg.de/cgi-bin/longevityrecords/entry.plx

http://redbook-ua.org

Mammals

Al-Agaili A. 2003. Bats of Saudi Arabia. Unpublished manuscript.

Alves PC, Goncalves H, Santos M, Rocha A. 2002. Reproductive biology of the Iberian hare, *Lepus granatensis,* in Portugal. Mammalian Biology 67: 358-371.

Amori G, Contoli L, Nappi A. 2008. Fauna d'Italia Vol. 44. Mammalia II. Erinaceomopha, Soricomorpha, Lagomorpha, Rodentia. Calderini, Bologna.

Angerbiorn A, Flux JEC. 1995. *Lepus timidus*. Mammalian Species 495: 1-11.

Aulagnier S, Haffner P, Mitchell-Jones T., Moutou F, Zima J. 2011. Guide del mammiferes d'Europe, d'Afrique du Nord et du Moyen-Orient. Delachaux & Niestlé SA, Paris.

Baron B, Borg JJ. 2011. Evidence of niche expansion in the *Myotis punicus* (*Mammalia Chiroptera*) of the Maltese islands. Naturalista Siciliano 35: 407-417.

Beuneux G. 2004. Morphometrics and ecology of *Myotis* cf. *punicus* (Chiroptera, Vespertilionidae) in Corsica. Mammalia 68: 269-273.

Bogdanowicz W. 1994. *Myotis daubentonii*. Mammalian Species 475: 1-9.

Boitani L, Lovari S, Vigna Taglianti A (eds). 2003. Fauna d'Italia Vol. 38. Mammalia III. Carnivora-Artiodactyla. Calderini, Bologna.

Buruldag E, Kurtonur C. 2001. Hibernation and postnatal development of the mouse-tailed dormouse, *Myomimus roachi* reared outdoor's in a cage. Trakya University Journal of Scientific Research Series B 2: 179-186.

Caroli L, Capizzi D, Luiselli L. 2000. Reproductive strategies and life-history traits of the Savi's pine vole, *Microtus savii*. Zoological Science 17: 209-216.

Churchfield S, Rychlik L, Yavrouyan E, Turlejski K. 2006. First results on the feeding ecology of the Transcaucasian water shrew *Neomys teres* (Soricomorpha: Soricidae) from Armenia. Canadian Journal of Zoolory 84: 1853-1858.

Clark HO, Murdoch JD, Newman DP, Sillero-Zubiri C. 2009. *Vulpes corsac* (Carnivora: Canidae). Mammalian Species 832: 1-8.

Colak E, Yigit N, Sozen M, Verimli R. 1999. A study on morphology and karyology of *Prometheomys schaposchnikowi* Satunin, 1901 (Mammalia: Rodentia) in Turkey. Turkish Journal of Zoology 23: 415-421.

Coskun Y, Uluturk S. 2003. Observations of the mole vole, *Ellobius lutescens* Thomas 1897, (Mammalia: Rodentia) in Turkey. Turkish Journal of Zoology 27: 81-87.

Danilkin AA. 1995. *Capreolus pygargus*. Mammalian Species 512: 1-7.

Darvish J, Akbary Rad S, Siahsar-Vie R, Hosein Pour Feizi MA, Ghorbani F. 2010. New record of pigmy field mice (Genus *Apodemus*, Muridae, Rodentia) from northeastern

Iran. Hystrix – Italian Journal of Mammalogy 21: 115-126.

DeMaster DP, Stirling I. 1981. *Ursus maritimus*. Mammalian Species 145: 1-7.

Feldhamer GA, Farris-Renner KC, Barker CM. 1988. *Dama dama*. Mammalian Species 317: 1-8.

Feoktistova NY, Naidenko SV, Surov AV, Menchinskii EM. 2013. Ecological and physiological characteristics of seasonal biology of the Mongolian hamster, *Allocricetulus curtatus* Allan 1940 (Cricetinae, Rodentia). Ekologiya 1: 60-64.

Franzmann AW. 1981. *Alces alces*. Mammalian Species 154: 1-7.

Garcia-Perea R, Ventura J, Lopez-Fuster MJ, Gisbert J. 1997. *Sorex granarius*. Mammalian Species 554: 1-4.

Ghorbani F, Darvish J, Gholi Kami H, Mirshamsi O. 2010. Rodent fauna of the western Golestan Province in northeast Iran. Iranian Journal of Animal Biosystematics 6: 37-48.

Golemanski V et al. 2007. Red data book of the Republic of Bulgaria. Volume 2 – Animals. http://e-ecodb.bas.bg/rdb/en/vol2/

Gorsuch WA, Larivière S. 2005. *Vormela peregusna*. Mammalian Species 779: 1-5.

Irwin NR, Speakman JR. 2003. Azorean bats *Nyctalus azoreum*, cluster as they emerge from roots, despite the lack of avian predators. Acta Chiropterologica 5: 185-192.

King CM. 1983. *Mustela erminea*. Mammalian Species 195: 1-8.

Kingswood SC, Blank DA. 1996. *Gazella subgutturosa*. Mammalian Species 518: 1-10.

Kivanc E, Mutlu Eyison H, Kiralp S, Ekim O. 2013. Reproductive biology of *Acomys cilicicus* Spitzenberger, 1978 (Rodentia: Muridae) in Turkey. Turkish Journal of Zoology 37: 133-142.

Koffler BR. 1972. *Meriones crassus*. Mammalian Species 9: 1-4.

Kwiecinshi GG, Griffiths TA. 1999. *Rousettus egyptiacus*. Mammalian Species 611: 1-9.

Krystufek B. 2008. *Glis glis* (Rodentia: Gliridae). Mammalian Species 42: 195-206.

Lanza B. 2012. Fauna d'Italia Vol. 47. Mammalia V. Chiroptera. Calderini, Bologna.

Larivière S, Pasitschniak-Arts M. 1996. *Vulpes vulpes*. Mammalian Species 537: 1-11.

Lopez-Fuster MJ, Ventura J. 1996. A morphometrical review of the *Sorex araneus-arcticus* species group from the Iberian peninsula (Insectivora, Soricidae). Bonner zoologische Beitrage 46: 327-337.

Lyman CP, O'Brien R, Green GC, Papafrangos ED. 1981. Hibernation and longevity in the Turkish hamster *Mesocricetus brandi*. Science 212: 668-670.

Macdonald DW, Barrett P. 2001. Mammals of Europe.

Metch LD. 1974. *Canis lupus*. Mammalian Species 37: 1-6.

Mitchell-Jones AJ et al. 1999. The atlas of European mammals. T & AD Poyser, London.

Ognev SI. 1950. Mammals of the USSR and adjacent countries. Vol. II Rodents. Izdatel'stvo Akedemii Nauk SSSR, Movska-Leningrad.

Ozkurt S, Yigit N. 2005. Observations on the ecology, reproduction and behavior of *Spermophilus* Bennett, 1835 (Mammalia: Rodentia) in Turkey. Turkish Journal of Zoology 29: 91-99.

Palmeirim JM, Hoffmann RS. 1983. *Galemys pyrenaicus*. Mammalian Species 207: 1-5.

Palomo LJ. 2008. Atlas y libro rojo de los mamiferos terrestres de espana. ICONA (Organismo Autonomo Parques Nacionales), Madrid.

Palomo LJ, Justo ER, Vargas JM. 2009. *Mus spretus* (Rodentia: Muridae). Mammalian Species 840: 1-10.

Parrini F, Cain JW, Krausman PR. 2009. *Capra ibex* (Artiodactyla: Bovidae). Mammalian Species 830: 1-12.

Pasitschaniak-Arts M. 1993. *Ursus arctos*. Mammalian Species 439: 1-10.

Pasitschaniak-Arts M, Larivière S. 1995. *Gulo gulo*. Mammalian Species 499: 1-10.

Rieger I. 1981. *Hyaena hyaena*. Mammalian Species 150: 1-5.

Rogovin KA. 1992. Habitat use by two species of Mongolian marmots (*Marmota sibirica* and *M. baibacina*) in a zone of sympatry. Acta Theriologica 37: 345-350.

Rydell J. 1989. Feeding activity of the northern bat *Eptesicus nilssoni* during pregnancy and lactation. Oecologia 80: 562-565.

Rydell J. 1993. *Eptesicus nilssonii*. Mammalian Species 430: 1-7.

Rydell J, Bogdanowicz W. 1997. *Barbastella barbastellus*. Mammalian Species 557: 1-8.

Sanchez-Garcia C, Alonso ME, Bartolomé DJ, Pérez JA, Larsen RT, Gaudioso VR. 2012. Survival, home range patterns, probable causes of mortality, and den-site selection of the Iberian hare (Lepus, Leporidae, Mammalia) on arable farmland in north-west Spain. Italian Journal of Zoology 79: 590-597.

Sempéré AJ, Sokolov VE, Danilkin AA. 1996. *Capreolus capreolus*. Mammalian Species 538: 1-9.

Sheffield SR, King CM. 1994. *Mustela nivalis*. Mammalian Species 454: 1-10.

Simeonovska-Nikolova DM. 2000. Strategies in open field behaviour of *Mus spicilegus* and *Mus musculus musculus*. Belgian Journal of Zoology 130: 115-120.

Simeonovska-Nikolova DM, Mehmed S. 2009. Behavior of mound-building mouse, *Mus spicilegus* during autumn-winter period in captivity. Biotechnology & Biotechnological Equipment 23 (special edition online): 180-183.

Sokolov VE. 1974. *Saiga tatarica*. Mammalian Species 38: 1-4.

Sokolov VE, Kotenkova EV, Michailenko AG. 1998. *Mus spicilegus*. Mammalian Species 592: 1-6.

Spagnesi M, De Marinis AM (eds). 2002. Mammiferi d'Italia. Quaderni di Conservazione della Natura 14. Ministero dell'Ambiente – Istituto Nazionale della Fauna Selvatica.

Spitzenberger F. 1978. Die stachelmaus von Kleinasien, *Acomys cilicicus* n. sp. (Rodentia, Muridae). Annalen des Naturhistorischen Museums in Wien 81: 433-446.

Tchabovsky A, Bazykin G. 2004. Females delay dispersal and breeding in a solitary gerbil, *Meriones tamariscinus*. Journal of Mammalogy 85: 105-112.

Tinnin DS, Dunnum JL, Salazar-Bravo J, Batsaikhan N, burt MS, Gardner SL, Yates TL. 2002. Contributions to the mammalogy of Mongolia, with a checklist of the species of the country. Faculty Publications from the Harold W. Manter Laboratory of Parasitology. Paper 36. http://digitalcommons.unl.edu/parasitologyfacputs/36

Ushakova MV, Feoktistova NY, Petrovskii DV, Gureeva AV, Naidenko SV, Surov AV. 2010. Hibernation in the Eversman hamster (*Allocricetulus eversmanni* Brandt, 1859) from the Saratov trans-Volga region. Povolzhskii Ekologicheskii Zhurnal 4: 415-422.

Vohralik V, Sofianidou TS, Frynta D. 1998. Reproduction in *Mus macedonicus* (Mammalia: Rodentia) in the Balkans. Bonner zoologische Beitrage 47: 283-292.

Wilson DE, Mittermeier RA (eds). 2009. Handbook of the mammals of the world. Volume 1. Carnivores. Lynx Edition, Barcelona.

Wilson DE, Mittermeier RA (eds). 2011. Handbook of the mammals of the world. Volume 2. Hoofed mammals. Lynx Edition, Barcelona.

Yavuz M, Oz M, Albayrak I. 2011. Karyological and some biological features of the *Microtus levis* (Miller, 1908) (Mammalia: Rodentia) at the eleven new localities on Taurus, West Mediterranean region in Turkey. Journal of Applied Biological Sciences 5: 23-28.

Yigit N, Colak E. 1998. Contribution to the geographic distribution of rodent species and ecological analyses of their habitat in Asiatic Turkey. Turkish Journal of Biology 22: 435-446.

Yigit N, Colak E, Colak R, Ozkan B, Ozkurt S. 2003. On the Turkish populations of *Dryomys nitedula* (Pallas, 1779) and *Dryomys laniger* Felten and Storch, 1968 (Mammalia: Rodentia). Acta Zoologica Academiae Scientiarum Hungaricae 49: 147-158.

Yigit N, Colak E, Gattermann R, Neumann K, Ozkurt S, Gharkheloo MM, Fritzsche P, Colak R. 2006. Morphological and biometrical comparisons of *Mesocricetus* Nehring, 1898 (Mammalia: Rodentia) species distributed in the Palearctic region. Turkish Journal of

Zoology 30: 291-299.

Yigit N, Kankilic T, Colak E. 2007. Reproductive biology and postnatal development of *Microtus rossiaemeridionalis* Ognev, 1924 (Mammalia: Rodentia) distributed in Turkey. Turkish Journal of Zoology 31: 287-294.

Yigit N, Markov G, Colak E, Kocheva M, Saygili F, Yuce D, Cam P. 2012. Phenotypic features of the '*Guentheri*' group vole (Mammalia: Rodentia) in Turkey and Southeast Bulgaria: evidence for its taxonomic detachment. Acta Zoologica Bulgarica 64: 23-32.

Youngman PM. 1990. *Mustela lutreola*. Mammalian Species 362: 1-3.

Web sites

http://www.ultimateungulate.com/Artiodactyla
www.iucnredlist.org
http://eol.org
www.arkive.org
www.vertebradosiberico.org
http://www.demogr.mpg.de/cgi-bin/longevityrecords/entry.plx
www.gerbil.info
www.agroatlas.ru
http://redbook-ua.org

Reptiles

Arakelyan M S, Dalielyan F D, Corti C, Sindaco R, Leviton A E 2011. Herpetofauna of Armenia and Nagorno-Karabakh. Society for the Study of Amphibians and Reptiles, Ithaca, New York, USA.

Baier F, Sparrow DJ, Wiedl HJ. 2013. The amphibians and reptiles of Cyprus. Chimaira Editions, Frankfurt, 362 pp.

Baran, I. and Atatür, M. K. 1998. Turkish Herpetofauna: Amphibians and Reptiles. Ministry of Environment of Turkey. .

Corti C, Capula M, Luiselli L, Sindaco R, Razzetti E (Eds.). 2011. Fauna d'Italia, vol. 45, Reptilia. Calderini, Bologna, 869 pp.

David P, Vogel G. 2010. Venemous snakes of Europe, Northern, Central, and Western Asia. Chimaira Editions, Frankfurt, 160 pp.

Disi AM, Modry D, Necas P, Rifai L. 2001. Amphibians and reptiles of the Hashemite Kingdom of Jordan. Chimaira Editions, Frankfurt, 408 pp.

El-Oran RM, Al-Melhem WN, Amr ZS. 1994. Snakes of southern Jordan. Italian Journal of Zoology 61: 359-367.

Gasc JP, Cabela A, Crnobrnja-Isailovic J, Dolmen D, Grossenbacher K, Haffner P, Lescure J, Martens H, Martínez Rica JP, Maurin H, Oliveira ME, Sofianidou TS, Veith M, Zuiderwijk A (Eds). 1997. Atlas of amphibians and reptiles in Europe. Collection Patrimoines Naturels, 29, Societas Europaea Herpetologica, Muséum National d'Histoire Naturelle & Service du Patrimoine Naturel, Paris, 496 pp.

Golemanski V et al. 2007. Red data book of the Republic of Bulgaria. Volume 2 – Animals. http://e-ecodb.bas.bg/rdb/en/vol2/

Khan MS 2006. Amphibians and reptiles of Pakistan. Krieger Publishing Company, Malabar, Florida.

Kreiner G. 2007. The snakes of Europe: all species from west of the Caucasus mountains. Chimaira Editions, Frankfurt, 317 pp.

Murphy R W, Fu J, Macculloch R D, Darevsky I S, Kupriyanova L A 2000. A fine line between sex and unisexuality: the phylogenetic constraints on parthenogenesis in lacertid lizards. Zoological Journal of the Linnean Society 130: 527-549.

Phelps T. 2010. Old world vipers: a natural history of the Azemiopinae and Viperinae. Chimaira Editions, Frankfurt, 558 pp.

Schleich HH, Kästle W, Kabish K 1996. Amphibians and reptiles of North Africa. Koeltz, Koenigstein, Germany.

Sindaco R, Doria G, Razzetti E, Bernini F (Eds). 2006. Atlas of Italian amphibians and reptiles. Societas Herpetologica Italica, Edizioni Polistampa, Firenze, 792 pp.

Valakos ED, Pafilis P, Sotiropoulos K, Lymberakis P, Maragou P, Foufopulos J. 2008. The amphibians and reptiles of Greece. Chimaira Editions, Frankfurt, 563 pp.

Web Sites

www.herpfrance.com

www.arkive.org

www.iucnredlist.org

http://eol.org

www.vertebradosiberico.org

http://www.demogr.mpg.de/cgi-bin/longevityrecords/entry.plx

http://redbook-ua.org

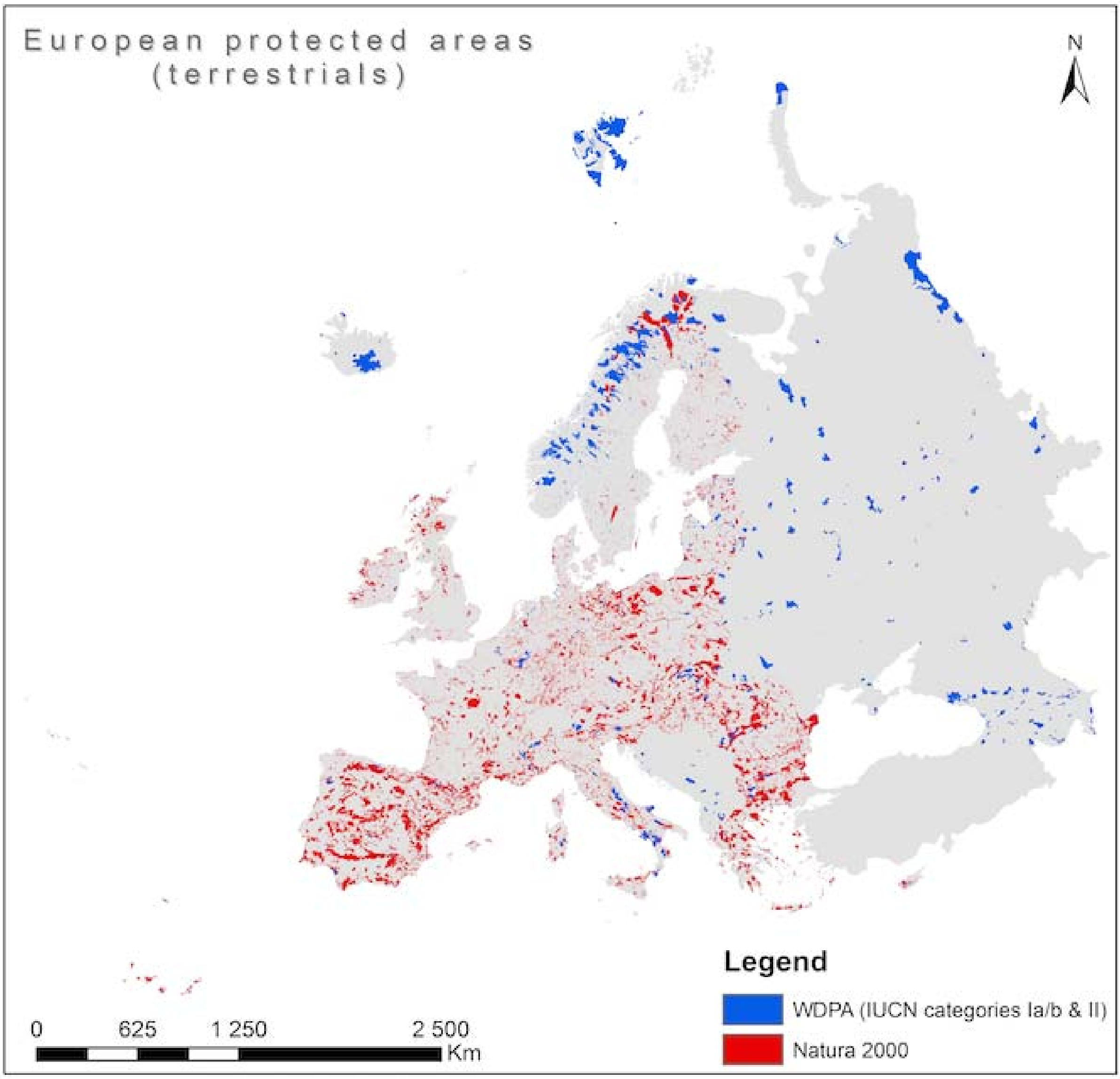

Figure S1. Distribution of protected areas in Europe. Only the protected areas considered in the study are mapped. They are represented in blue by PAs belonging to IUCN category I and II from the World Database on Protected Area (WDPA, http://protectedplanet.net/) and in red by the complete Natura 2000 areas (http://www.eea.europa.eu/) for the EU28 belonging to the entire European sub-continent.

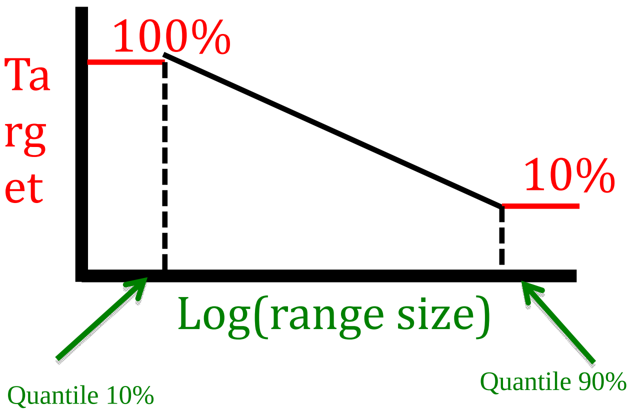

Figure S2 Target definition for the four groups. For each group, we extracted the species for which species range size belonged to the top 10 and 90% quantiles. For those species, the target was fixed to 100% and to 10%. In order words, a target of 100% was set for the 10% rarest species of each group, and the target of 10% was set for the 10% most common species. For the remaining species, a regression was fitted between the coordinates (10,100) and (90,10) to define their respective targets. Species target achievement was then measured at the ratio between the percentage of range actually covered by the PA system and the target.

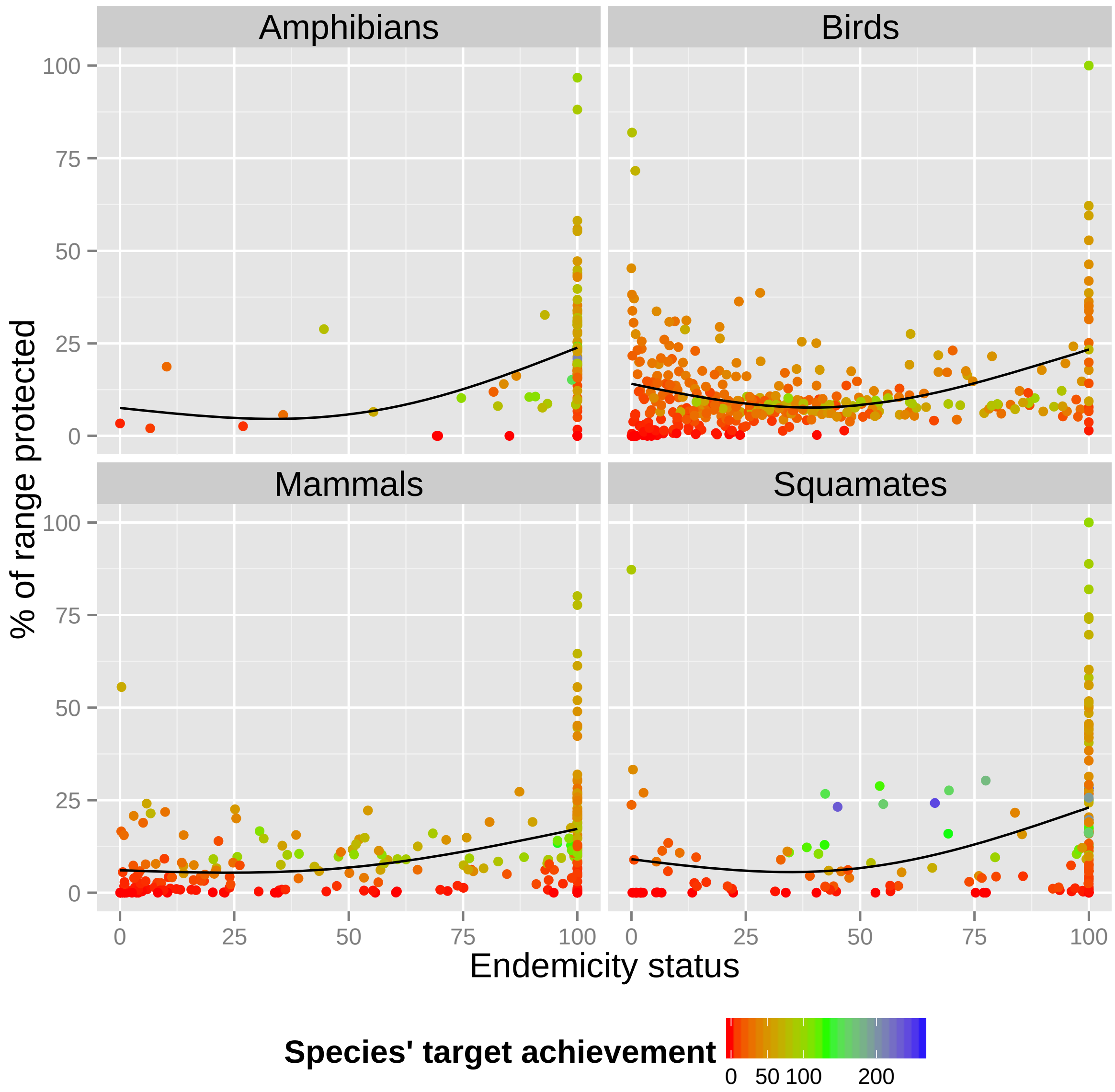

Figure S3 Percentage of range of protected in Europe in function of the endemicity status (% of global range encapsulated in the European region under study). Species' target achievement is also indicated using a colour gradient from red to blue (low to high). When endemicity equals to 100%, it means the species is a strict endemic of the European region under study.

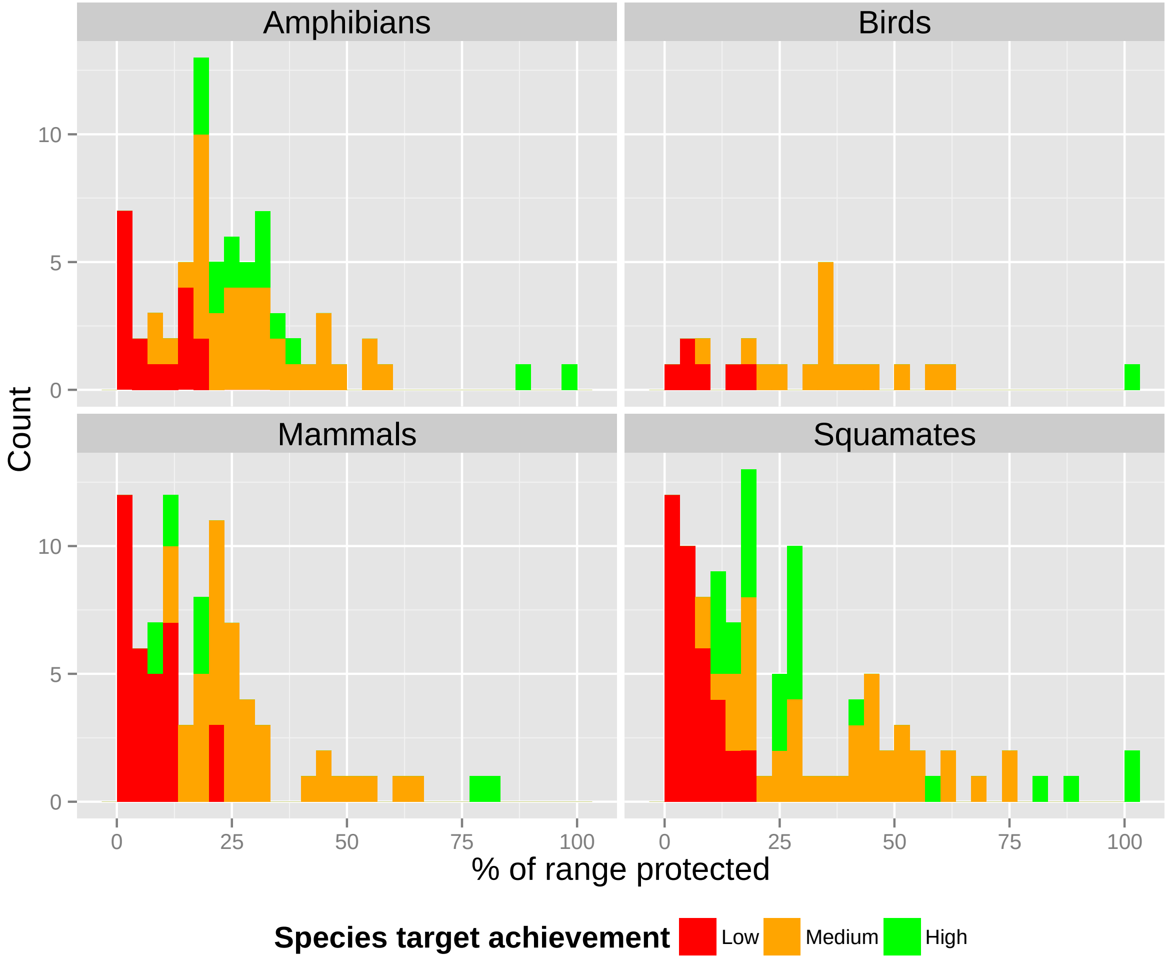

Figure S4. **Percentage of range protected for endemic species in Europe.** The Y-axis represents a number of species (over a total of 280 species) and X-axis the percentage of range currently protected in Europe. The filled bars represent the species' target achievements in three classes, namely low, medium and high, when species' target achievement was lower than 25%, between 25 and 75% and higher than 75%, respectively.

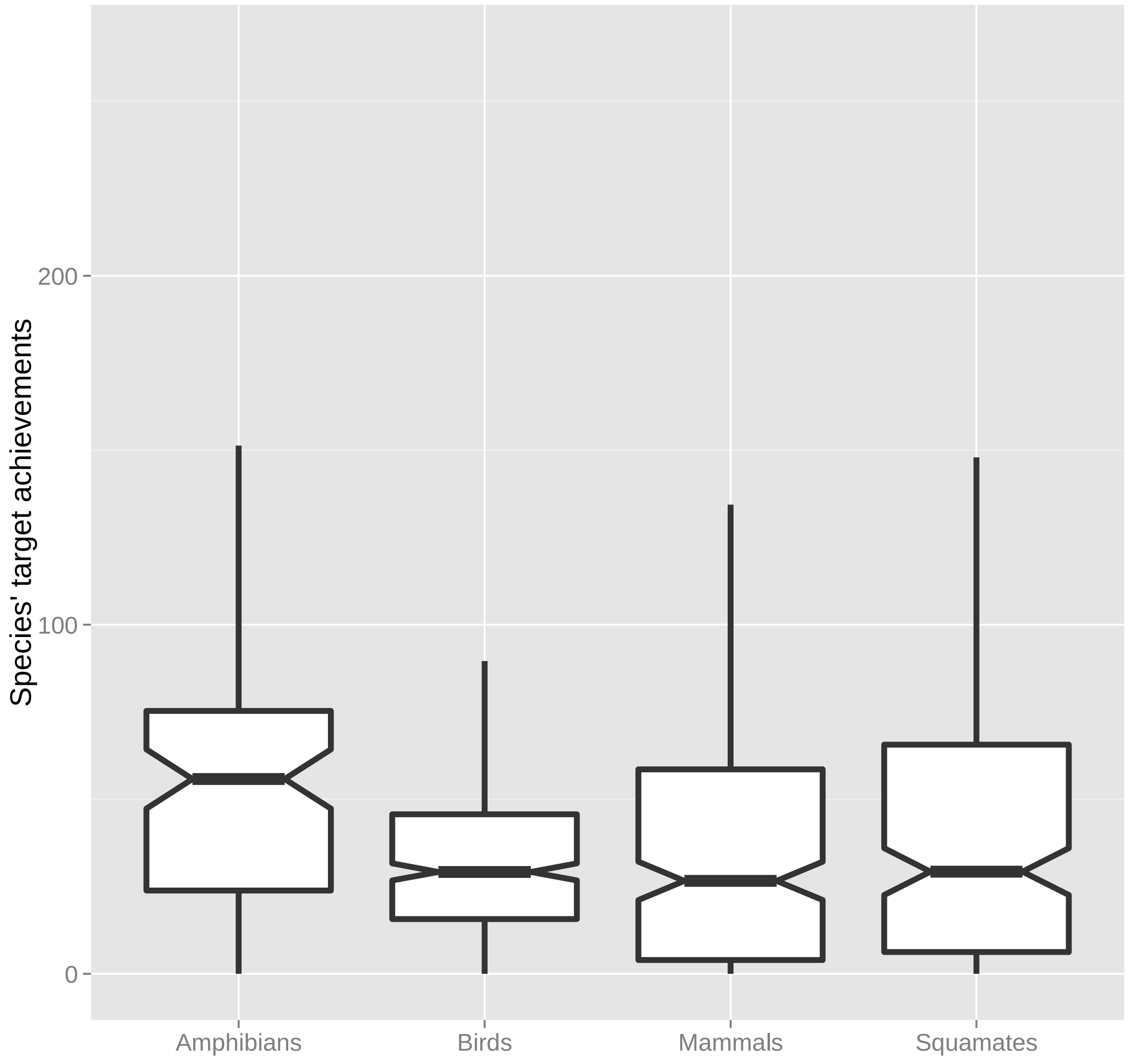

Figure S5. **Species' target achievement across the four groups**. The outliers are not displayed. The upper and lower "hinges" correspond to the first and third quartiles (the 25th and 75th percentiles). The notches give a roughly 95 interval for comparing medians. Amphibians have significantly higher species' target achievements that the other three groups.

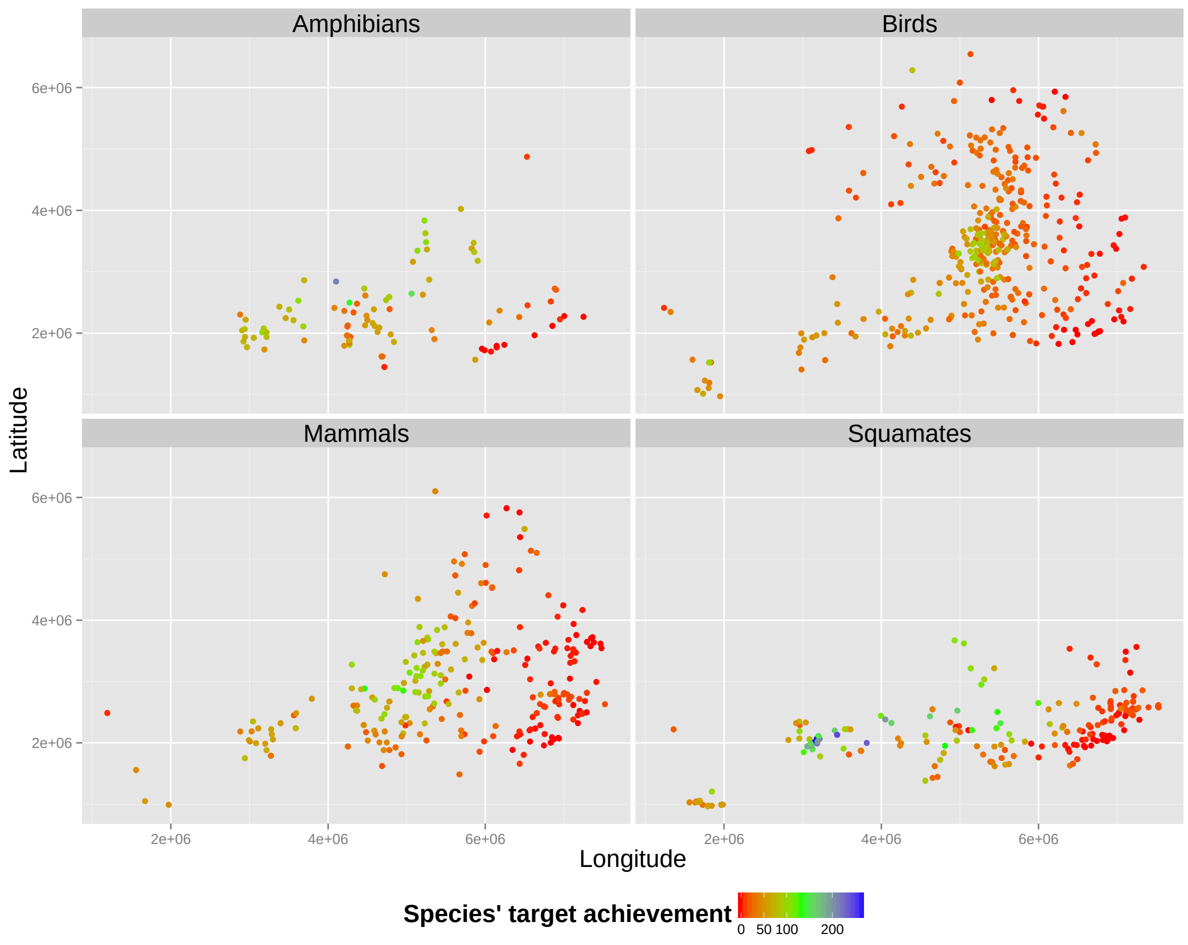

Figure S6 Mean latitude and longitude of all species in function of species' target achievement.

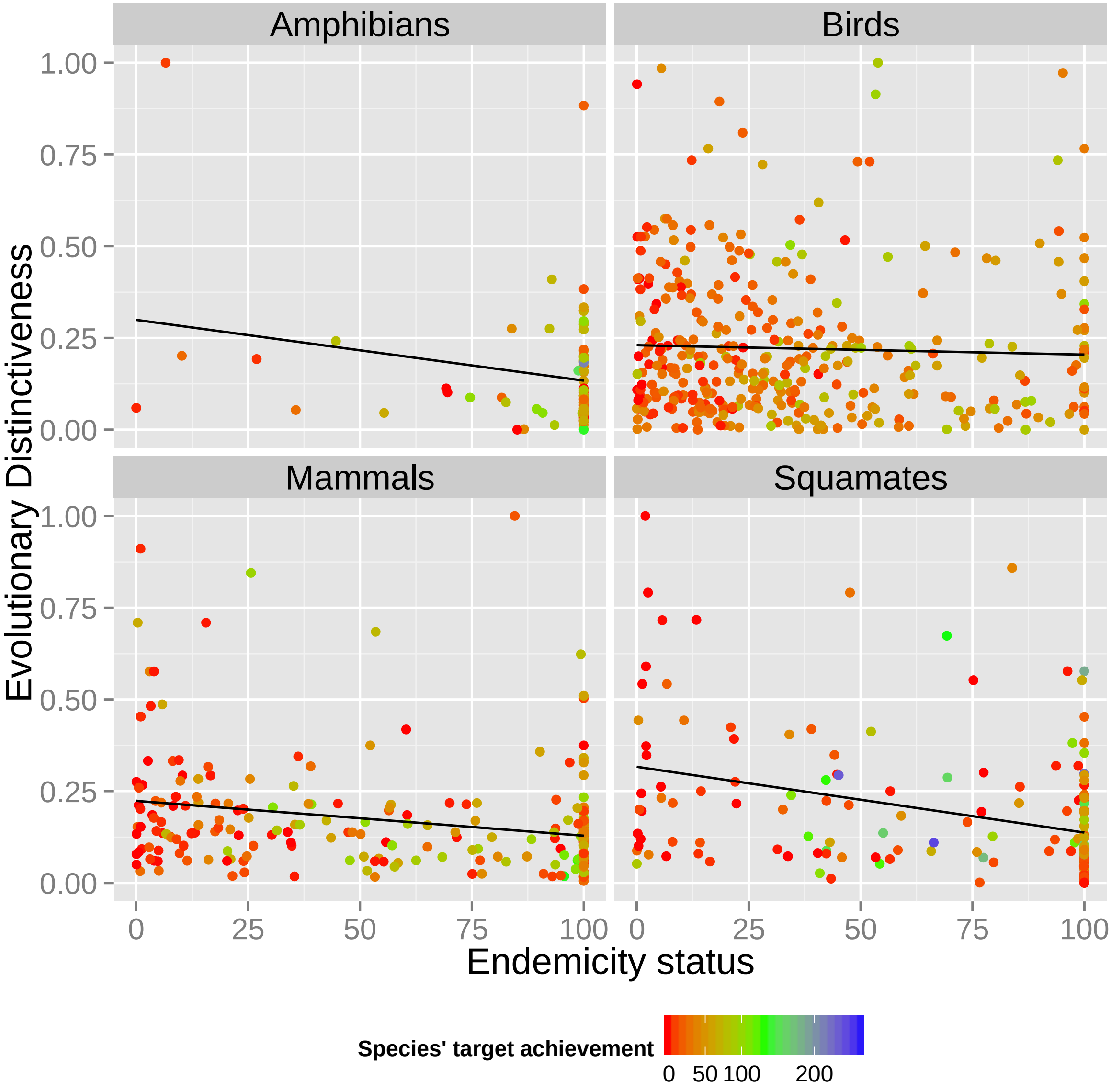

Figure S7. Evolutionary distinctiveness in function of endemicity status (% of global range encapsulated in the European region under study). When endemicity equals to 100%, it means the species is a strict endemic of the European region under study.

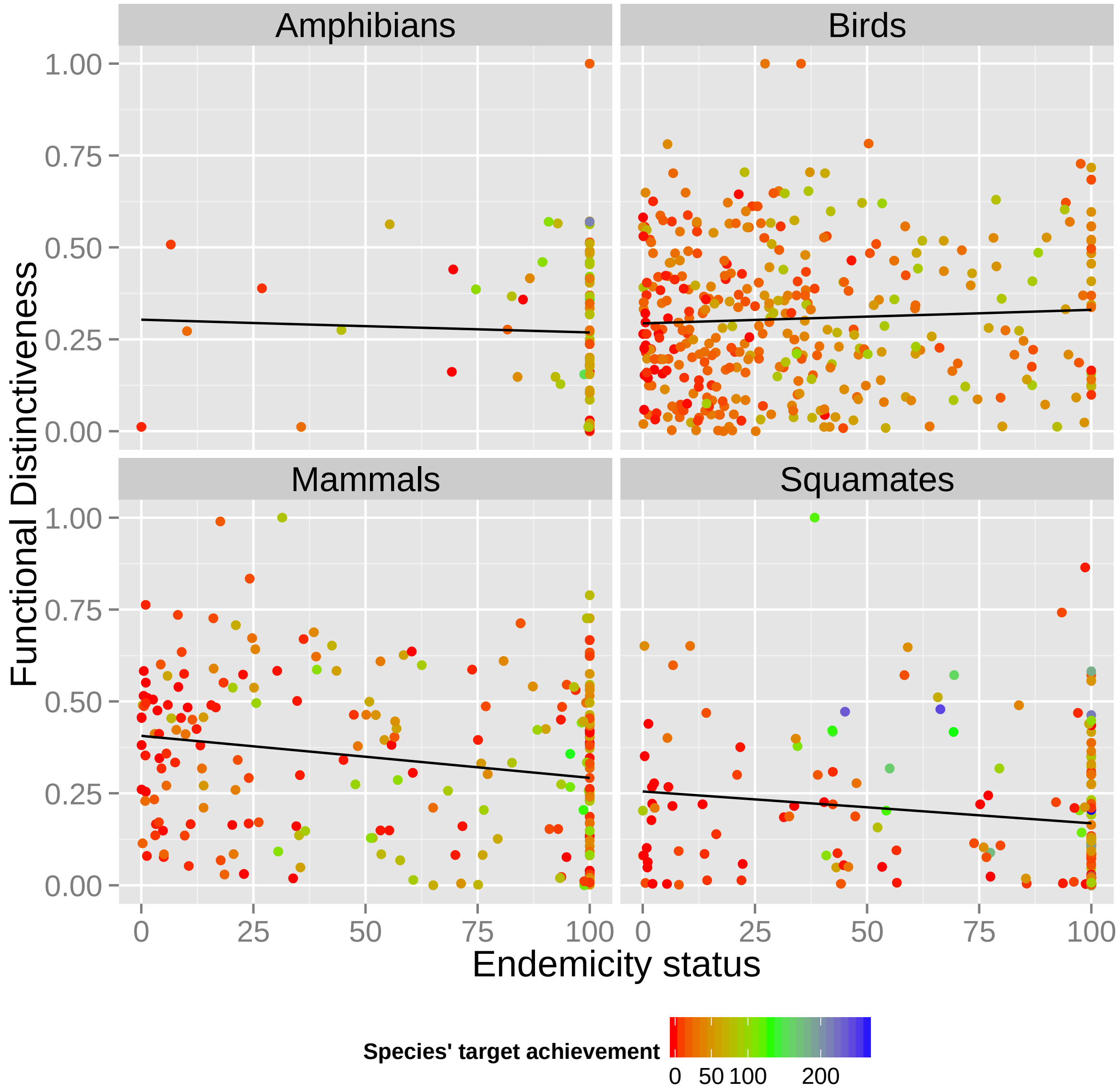

Figure S7. Functional distinctiveness (Y-axis) in function of percentage of global range encapsulated in Europe (endemicity status, X-axis). When endemicity equals to 100%, it means the species is a strict endemic of the European region under study.

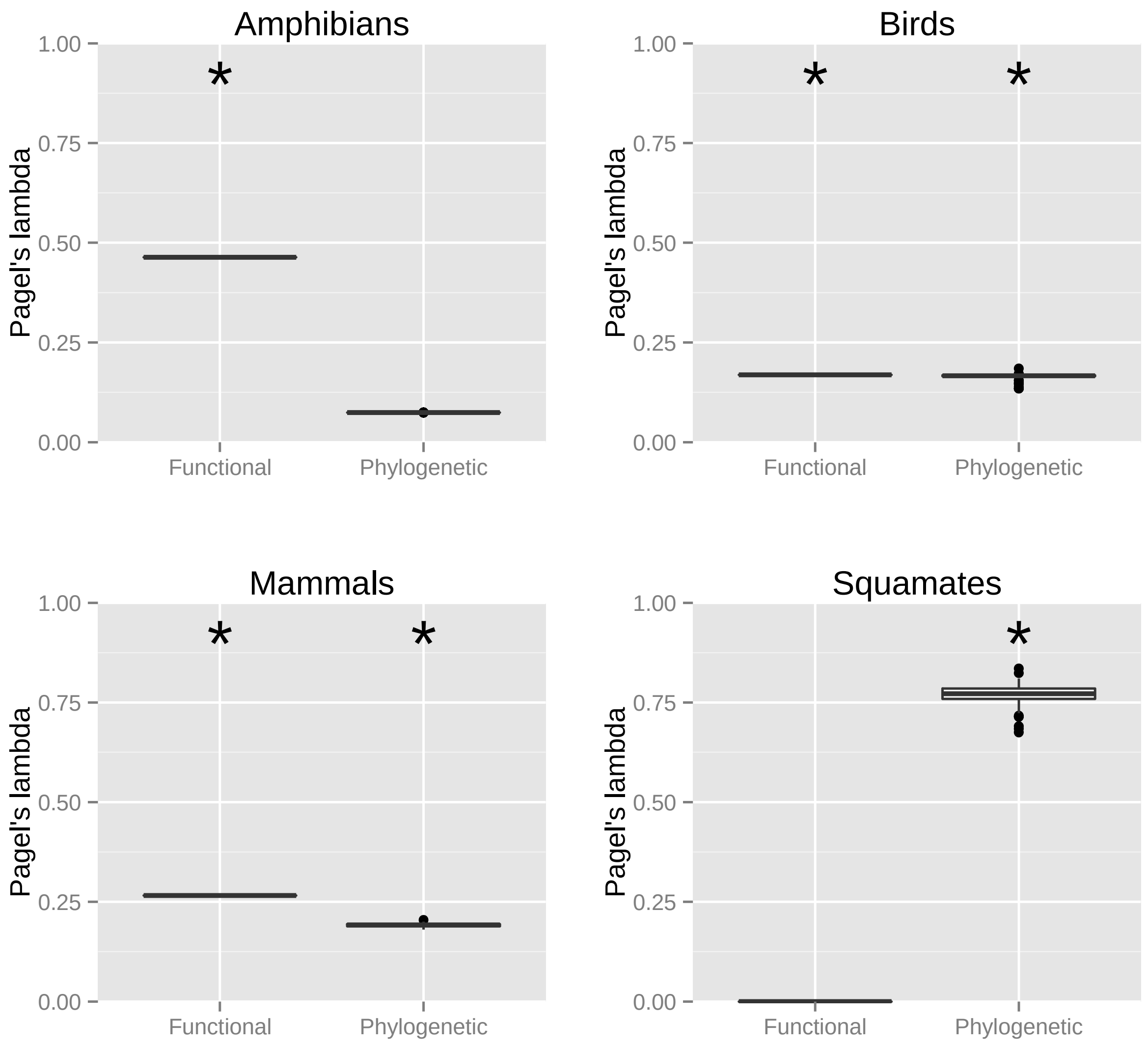

Figure S9. **Phylogenetic and functional signals of species' target achievement**. We used a maximum-likelihood based measurement of phylogenetic/functional signal, namely lambda model, as developed by [2]. This metric corresponds to a tree transformation parameter, which gradually eliminates phylogenetic/functional structure when varying from 1 to 0. Lambda transformation is performed by multiplying the off-diagonal elements of the variance/covariance matrix describing the tree topology and branch lengths. Lambda values of 1 correspond to a Brownian evolution, whereas at the other extreme a lambda value of 0 corresponds with the complete absence of phylogenetic/functional structure (star-like phylogeny). The estimated lambda can be compared to zero by computing a likelihood ratio and comparing it to a chi-square distribution with one degree of freedom. Hence testing for a significant phylogenetic signal, relative to phylogenetically unstructured data. The stars on the top of each barplot correspond to a signal significantly different from 0. For the phylogenetic

signal, we use False Discovery Rate to estimate the phylogenetic signal across the 100 trees.

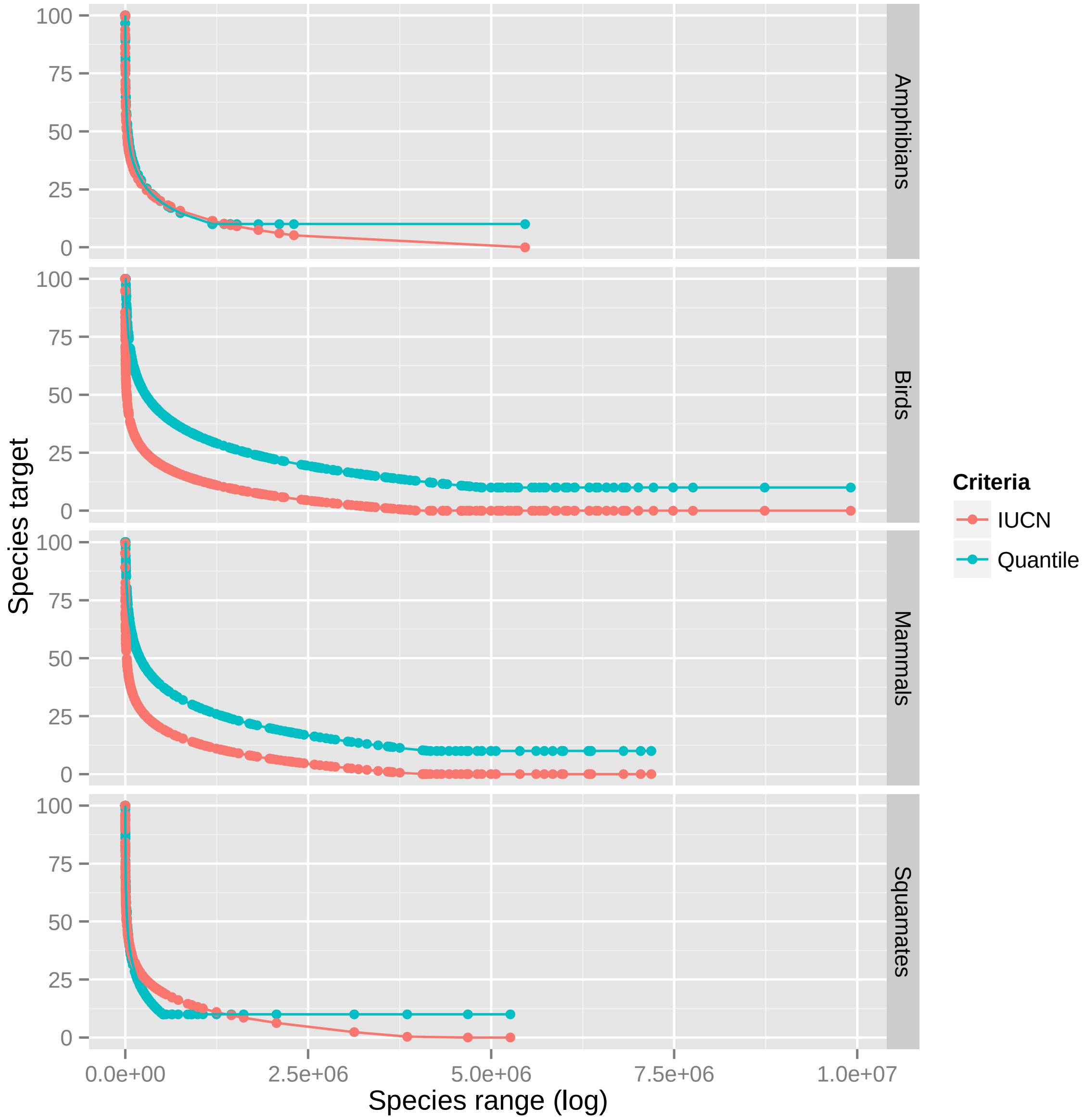

Figure S10 Relationship between species' targets and species ranges in function of the criteria used. The "quantile" approach is the one used in the main text (Figure S1). The second one used IUCN categories. We used the criteria B1 based on species range to define critically endangered species, species with less than 100km^2 range and vulnerable for species with less than 10,000km^2. We arbitrarily set that critically endangered species should be protected at 100% and vulnerable at 50%. We then used a regression along these two points to set species targets for all species. Although the ICUN approach is not group-specific the species targets are pretty similar to the ones defined through the quantile approach. Only birds and mammals do differ meaning that our Quantile approach is more severe than the IUCN one (i.e. the quantile approach asks for larger targets meaning high species' target achievement are more difficult to reach).

Table S1. *P-value* of the OLS regression lines of figure 2 and 3 and the associated r-squares of the regressions.

		Evolutionary distinctiveness	Functional distinctiveness
**Amphibian**	*p-value*	0.8	**0.01**
	R^2	0.0007	0.066
**Birds**	*p-value*	0.79	0.29
	R^2	0.0002	0.003
**Mammals**	*p-value*	0.46	0.27
	R^2	0.002	0.004
**Squamates**	*p-value*	0.56	**0.049**
	R^2	0.002	0.02

Table S2. Representation of the land cover types in Europe and their representation in the European protected area network. A pixel has a resolution of 300m. Only the total number of pixels of each land cover type is represented (No of pixels), as well as the number of pixels that fall within a protected area (No of pixels within PAs) and the associated percentage of protection (% in PA)

11	Post-flooding or irrigated croplands (or aquatic)	2782	59	2
13	Post-flooding or irrigated herbaceous crops	170896	10485	6
14	Rainfed croplands	16934587	746667	4
15	Rainfed herbaceous crops	199	31	16
16	Rainfed shrub or tree crops (cash crops, vineyards, olive tree, orchardsà)	23959	737	3
20	Mosaic cropland (50-70%) / vegetation (grassland/shrubland/forest) (20-50%)	17886778	740541	4
21	Mosaic cropland (50-70%) / grassland or shrubland (20-50%)	2093	42	2
30	Mosaic vegetation (grassland/shrubland/forest) (50-70%) / cropland (20-50%)	7894080	53178	1
32	Mosaic forest (50-70%) / cropland (20-50%)	6420	1273	20
40	Closed to open (>15%) broadleaved evergreen or semi-deciduous forest (>5m)	38	6	16
41	Closed (>40%) broadleaved deciduous forest (>5m)	1655	132	8
50	Closed (>40%) broadleaved deciduous forest (>5m)	21609742	1803096	8
60	Open (15-40%) broadleaved deciduous forest/woodland (>5m)	701	34	5
70	Closed (>40%) needleleaved evergreen forest (>5m)	3464303	791684	23
90	Open (15-40%) needleleaved deciduous or evergreen forest (>5m)	8819597	571849	6
91	Open (15-40%) needleleaved deciduous forest (>5m)	101	1	1
92	Open (15-40%) needleleaved evergreen forest (>5m)	6442376	461757	7
100	Closed to open (>15%) mixed broadleaved and needleleaved forest (>5m)	13182533	698442	5
101	Closed (>40%) mixed broadleaved and needleleaved forest (>5m)	18668	3418	18
110	Mosaic forest or shrubland (50-70%) / grassland (20-50%)	4677509	432529	9
120	Mosaic grassland (50-70%) / forest or shrubland (20-50%)	664653	87181	13
130	Closed to open (>15%) (broadleaved or needleleaved, evergreen or deciduous) shrubland (<5m)	1877892	339533	18
131	Closed to open (>15%) broadleaved or needleleaved evergreen shrubland (<5m)	9231	2611	28
134	Closed to open (>15%) broadleaved deciduous shrubland (<5m)	406326	40537	10
140	Closed to open (>15%) herbaceous vegetation (grassland, savannas or lichens/mosses)	669622	65468	10

141	Closed (>40%) grassland	2350065	148420	6
143	Open grassland	1853	148	8
150	Sparse (<15%) vegetation	21618525	2177227	10
151	Sparse (<15%) grassland	2049257	30026	1
152	Sparse (<15%) shrubland	802712	106979	13
180	Closed to open (>15%) grassland or woody vegetation on regularly flooded or waterlogged soil - Fresh, brackish or saline water	2848224	233475	8
190	Artificial surfaces and associated areas (Urban areas >50%)	1097134	27652	3
200	Bare areas	1239330	198848	16
201	Consolidated bare areas (hardpans, gravels, bare rock, stones, boulders)	13478	6146	46
202	Non-consolidated bare areas (sandy desert)	3548	2058	58
203	Salt hardpans	791	356	45
210	Water bodies	4302439	553591	13
220	Permanent snow and ice	5256077	1647068	31
230	No data (burnt areas…)	526	16	3
190	Artificial surfaces and associated areas (Urban areas >50%)	1097134	27652	3

[1] Pearman, P.B., Lavergne, S., Roquet, C., Wuest, R., Zimmerman, N.E. & Thuiller, W. 2014 Phylogenetic patterns of climatic, habitat and trophic niches in a European avian assemblage. *Global Ecology and Biogeography*.
[2] Pagel, M.D. 1997 Inferring evolutionary processes from phylogenies. *Zoologica Scripta* **26**, 331-348.
